# Exploring circulating cell-free DNA as a biomarker and as an inducer of AIM2-inflammasome-mediated inflammation in patients with abdominal aortic aneurysm

**DOI:** 10.1038/s41598-025-06220-5

**Published:** 2025-06-20

**Authors:** Susanne Dihlmann, Carolin Kaduk, Karola H. Passek, Anja Spieler, Dittmar Böckler, Andreas S. Peters

**Affiliations:** 1 Department of Vascular and Endovascular Surgery, University Hospital Heidelberg, Im Neuenheimer Feld 420, 69120 Heidelberg, Germany; 2 Vaskuläre Biomaterialbank Heidelberg (VBBH), Im Neuenheimer Feld 420, 69120 Heidelberg, Germany; 3 Department of Vascular and Endovascular Surgery, University Hospital Frankfurt, Theodor-Stern-Kai 7, 60590 Frankfurt Am Main, Germany

**Keywords:** Cell free DNA, mtDNA, Abdominal aortic aneurysm, AIM2, Inflammasome, Biomarker, Proinflammatory response, THP-1 cells, Cell biology, Molecular biology, Biomarkers, Medical research, Molecular medicine, Pathogenesis

## Abstract

Circulating cell-free (cf) DNA in blood plasma is considered a diagnostic and prognostic biomarker of tissue damage and could be a driver of chronic inflammation by stimulating the innate immune response via activation of inflammasomes. Increased AIM2-inflammasome activity in the aortic wall is associated with abdominal aortic aneurysm (AAA). We here hypothesized that cfDNAs are elevated in the plasma of AAA patients and are associated with chronic inflammation. Single strand (ss)DNA, double strand (ds)DNA and mitochondrial (mt)DNA levels were explored in plasma and leucocytes from 93 AAA patients, 89 controls (non-AAA patients) and 10 healthy subjects, using fluorescence-based quantification and real-time qPCR, respectively. To analyse inflammasome activation by cfDNA, differentiated THP-1 macrophages were primed with lipopolysaccharide (LPS) and then stimulated for one, six or 24 h with DNA extracted from peripheral blood mononuclear cells (PBMC) of AAA patients. Our analysis revealed significantly increased levels of ssDNA, dsDNA and mtDNA levels in plasma from AAA patients compared with non-AAA patients and healthy subjects. In addition, the mtDNA copy number was significantly higher in PBMC from AAA patients. Stimulation of THP-1 cells with PBMC-DNA resulted in increased expression of inflammasome genes, especially the DNA sensors *AIM2* and *IFI16*. At early time points, PBMC-DNA stimulated THP-1 showed significantly increased apoptosis-associated speck-like protein with a CARD (ASC) and Pro-Interleukin-1β protein levels compared to untreated or only LPS-primed cells, resulting in the formation of significantly more ASC specks after 24 h, a sign of inflammasome activation. We conclude from our data that cfDNA of AAA patients triggers a proinflammatory response in macrophages by activating the AIM2 inflammasome and thus could be a driving force for the chronic inflammation observed in these patients.

## Introduction

Circulating cell-free (cf) DNA possesses considerable potential for the study of pathological conditions in patients with different disease statuses^1,2^. Accordingly, an increasing number of studies aim to use cfDNA as a biomarker for different clinical applications (for review see^3^). CfDNA consists of short DNA fragments that originate from both nuclear DNA (nDNA) and extrachromosomal mitochondrial DNA (mtDNA), and are released into the bloodstream in the course of apoptosis, necrosis or by active secretion from cells, i.e. neutrophils^4^. In healthy individuals, the majority of circulating nDNA fragments extracted from plasma is between 70 and 220 bp long and packed in mononucleosomes or nucleosome/chromatosome particles, protecting it from blood DNases. About 9% of the cell-free nDNA was recently found to be associated with apoptotic bodies, cell debris or large extracellular vesicles, and about 28% were found to be bound in chromatin or associated with small extracellular vesicles^5^. The appearance of mtDNA in plasma is contradictory. While some authors reported mtDNA to be degraded in short fragments of less than 100 bp^6^, others reported that mtDNA in plasma is poorly fragmented, appearing longer than 1000 bp, which suggests stabilizing factors, despite the lack of histones^5^. The latter described mtDNA to be in about 76% associated with extracellular mitochondria, either in its free form or with large extracellular vesicles. A lesser extent of mtDNA was detected to be associated with small extracellular vesicles, exosomes or protein complexes^5^.

The tissue source of cfDNA in healthy controls varies from individual to individual, with approximately 70% of cfDNA produced by hematopoietic cells. However, the tissue source, its composition and physical characteristics change significantly with disease or disease state and may thus be used as a diagnostic marker or for monitoring disease progression^4^. Because cfDNA is released from apoptotic and necrotic cells, it has become particularly useful to monitor progression of tissue degeneration for example in organ transplantation^7^, in cancer and in cardiovascular diseases (CVD)^3,4^. However, the involvement of circulating cfDNA in progression and inflammatory processes of abdominal aortic aneurysms (AAA) has not yet been investigated, in detail.

AAA is characterized by a progressive dilation of all layers of the aortic wall^8^. Without surgical intervention, 50–70% of aortic dilations that were small and asymptomatic in the first clinical presentation progress until rupture, which is a life-threatening condition^9^. Small aneurysms are usually monitored by repeated imaging, such as ultrasound examinations. Surgical intervention by open or endovascular repair is recommended when the maximal diameter of the AAA reaches the intervention threshold (5.5 cm in male or 5.0 cm in female patients), or when it becomes symptomatic^10,11^. However, AAA present with varying risks of rupture, and patient-specific factors influence the stability of the aortic wall during disease progression. Despite extensive research, no biomarker is available to date that has sufficient sensitivity, specificity, and clinical validation to monitor progression of AAA and the individual rupture risk. Promising candidates for such biomarkers may be derived from the pathophysiology of AAA, which is characterized by aortic inflammation, formation of a large intra-luminal thrombus and remodelling of the aortic wall. The extensive cell-turnover in the AAA wall is expected to be associated with the release of necrotic cell debris, including cfDNA into the circulation. In addition, neutrophil extracellular traps (NET), a host defence mechanism which is significantly associated with the formation of AAA^12,13^, may result in accumulation of different cfDNAs in the blood. We therefore set out to investigate the diagnostic value of three different cfDNAs in plasma from AAA patients: nuclear single-strand (ss)DNA and double-strand (ds)DNA as well as mtDNA.

In addition to its diagnostic role as a potential biomarker, the contribution of circulating cfDNA to pathogenic mechanisms driving the progression of AAA is moving into research focus. For example, cfDNA plays a direct role in arterial and venous thrombosis^12^. Moreover, there is increasing evidence that different cfDNA types trigger inflammation in CVD by acting as damage associated molecular patterns (DAMP), which induce an innate immune response via inflammasomes in monocytes, macrophages or dendritic cells^14–16^. Inflammasomes are large cytoplasmic multi-protein complexes, bridging the recognition of pathogens or sterile damage to pyroptotic cell death and Il-1β driven inflammation. In particular, the AIM2 inflammasome can sense DNA derived from necrotic and apoptotic neighbouring cells^4^ as well as mtDNA released into the cytosol^17^.

We have previously detected increased AIM2 inflammasome expression in human AAA tissues^18,19^, in cell debris-challenged vascular smooth muscle cells^20^ and in PBMC from AAA patients^21,22^, and demonstrated a role for AIM2 in a mouse model for aortic aneurysm^23^. Therefore, we hypothesized that circulating cfDNAs may trigger AIM2 inflammasome activity in AAA, thereby contributing to AAA progression.

Considering the dual role of cfDNA as a potential biomarker and trigger of progression in various diseases, we here addressed both the levels of cfDNAs in plasma from AAA patients and the potential of different DNA types to induce an inflammatory response in macrophages. According to our data, cell-free ssDNA, dsDNA and mtDNA were elevated in plasma from AAA patients. In addition, the mtDNA copy number was significantly increased in PBMC from AAA patients. DNA samples isolated from PBMC of AAA patients induced mRNA expression of several inflammasome genes and corresponding proteins in THP1 macrophages, as well as the formation of ASC specks, indicating inflammasome activation.

## Materials and methods

### Blood samples and preanalytics

The study was conducted according to the Code of Ethics of the World Medical Association (Declaration of Helsinki). Patients undergoing treatment at the Klinik für Gefäßchirurgie und endovaskuläre Chirurgie Heidelberg were enrolled between January 2014 and December 2022. In addition, healthy volunteers were recruited between October 2022 and February 2023. Patients demographics were recorded by the Hospital Information System. Maximum aortic diameter of AAA patients was determined by computed tomography angiography scan. Aortic diameter of control patients was determined by routine ultrasound examination. Peripheral venous blood was taken from study participants on the day of their hospitalization into lithium-heparin (Sarstedt, Germany, S-Monovette, Lithium-Heparin-Gel) or Potassium-EDTA (Sarstedt, Germany, S-Monovette, EDTA KE) containing tubes, according to the standard operating procedures of the Vascular Biobank Heidelberg (VBBH). All participants gave their written informed consent to the study, which was approved by the ethical committee of the University of Heidelberg (S-091/2021) and (S-310/2013 “Etablierung einer humanen vaskulären Biomaterialbank (VBBH) an der Klinik für Gefäßchirurgie und endovaskuläre Chirurgie der Universitätsklinik Heidelberg), and amendments. The blood was processed for further studies within 4 h after venepuncture. Peripheral blood derived mononuclear cells (PBMC) were isolated by density gradient centrifugation using Ficoll-Paque Plus (Cytivia, Merck) as previously described^24^. Cells were counted using a Neubauer counting chamber, pelleted by centrifugation, and 5 × 10^6^ PBMC were resuspended in 200 µl PBS. Plasma was collected from Lithium-heparin containing tubes by centrifugation at 1200 g for 15 min. and aliquoted for further analysis. Plasma and PBMC (5 × 10^6^ cells/200 µl PBS) were stored at -80°C by the VBBH until used for the study.

### Diagnostic study design

An observational case–control study was conducted with blood plasma and PBMC samples, provided by the Vascular Biomaterial Bank Heidelberg (VBBH). Ninety-three samples from patients who underwent early diagnostics or treatment for repair of their abdominal aortic aneurysm (AAA) and 89 samples from patients without previous or present AAA (controls) were selected according to the following criteria: over 18 years old and informed consent of the patient to the study. Exclusion criteria for patients and controls were: recent (< 1 year) tumour and/or chemotherapy, systemic autoimmune or inflammatory disease. AAA was defined as a dilation of a maximal aortic diameter of ≥ 30 mm, affecting all layers of the aortic wall. Control samples were derived from vascular patients, who were admitted to the hospital for treatment (peripheral artery disease (PAD), thrombendarterectomy of the Arteria carotis interna (ACI), other thrombendarterectomies, minor vascular interventions, other), with a maximal aortic diameter of < 30 mm (screened by routine ultrasound examination) and no previous AAA or other aneurysms. AAA and control samples were matched in sex, age, BMI, and smoking habits. The patients’ characteristics are summarized in Table 1.

**Table 1:** Patient and control demographics and co-morbidities.

Characteristic	AAA (N = 93) Median (Range)	Non-AAA (N = 89) Median (Range)	*P*-value
Age (years)	70 (46–88)	71 (29–85)	0.8227
Body mass index (kg/m^2^)	26.6 (17.7–41.0)	25.9 (20.4–43.0)	0.1052

	N (%)	N (%)	
Sex			
Female	18 (19.35%)	27 (30.34%)	0.1214
Male	75 (80.65%)	62 (69.66%)	
Smoker status			
Current	30 (32.26%)	19 (21.35%)	0.2482
Ever	39 (41.94%)	41 (46.07%)	
Never	23 (24.73%)	29 (32.58%)	
Unknown	1 (1.1%)	0 (0%)	
Hypertension	84 (90.32%)	79 (88.76%)	0.8108
Antihypertensive therapy	84 (90.32%)	78 (87.64%)	0.6391
Hyperlipidaemia	80 (86.02%)	79 (88.76%)	0.6583
Lipid-lowering therapy	79 (84.95%)	78 (87.64%)	0.6696
PAD	31 (33.33%)	11 (11.83%)	0.0007
Antiplatelet therapy	69 (74.19%)	76 (85.39%)	0.0675
Coronary heart disease	56 (60.22%)	50 (56.18%)	0.6525
Myocardial infarction	30 (32.26%)	25 (28.09%)	0.6286
Stroke	11 (11.83%)	15 (16.85%)	0.3989
Diabetes	19 (20.43%)	32 (35.96%)	0.0217
Metformin therapy	10 (10.75%)	23 (25.84%)	0.0117
COPD	13 (13.98%)	4 (4.49%)	0.0398
Chronic kidney disease	18 (19.35%)	21 (23.60%)	0.5883
AAA family history	7 (7.69%)	0 (0%)	0.0139

AAA: abdominal aortic aneurysm; PAD: peripheral artery disease; COPD: chronic obstructive pulmonary disease; IQR: Interquartile range. Statistics: Mann Whitney test was used for continuous variables and Chi-Square test or Fisher’s exact test was used for dichotomous variables.

### Reagents and equipment

Names, providers and identifiers of reagents and equipment used throughout the study are listed in supplemental Tables 1 to 6.

### DNA isolation from plasma and PBMC

Frozen heparin-plasma samples, and lysates of 5 × 10^6^ PBMC in 200 µL PBS (derived from corresponding patients) were gently thawed in an ice bath. The plasma was centrifuged at 2500 g at 4° C for 15 min to eliminate remaining platelets from the plasma. Next, the supernatant (200 µl purified plasma) or the 200 µL PBS-PBMC suspension were transferred into fresh, 1.5 mL microreaction tubes. Once these samples reached room temperature, they were processed with the "DNeasy Blood & Tissue" kit (Qiagen, Germany) using the protocol: "Purification of Total DNA from Animal Blood or Cells (Spin-Column Protocol)”. In each case, 200 µL of plasma, or 200 µL of PBS containing 5 × 10^6^ PBMC lysate, was used as starting material. Following the protocol of the manufacturer, 20 µl Proteinase K (kit component) and 200 µl buffer AL (kit component) was added to each sample. The following procedures were performed exactly according to the instructions of the manufacturer. At the final step, DNA was eluted in 200 µl nuclease-free water.

### Quantitation of ssDNA and dsDNA from plasma and PBMC

The QuantiFluor ssDNA system and the QuantiFluor dsDNA system, respectively were used for sensitive quantification of nuclear DNA from plasma and PBMC samples. All procedures were performed according to the instructions of the manufacturer, using the multiwell plate protocol. Briefly, a standard curve was prepared using dsDNA or ssDNA standards (provided in the kit). Next, 200 µl of the QuantiFluor dsDNA or ssDNA working solution was added to each well in a 96 well plate and 10 µl of the prepared standards was added in duplicate to columns 1 and 2 of the plate. For analysis, 2–10 µl of unknown samples was added to the remaining wells in duplicate and the dilution factors were recorded for later calculation. Plates were mixed and incubated for 5 min. at room temperature, before the fluorescence was measured in a TECAN Spark multiplate reader.

### Quantitation of mtDNA by real-time qPCR

Plasma and PBMC mtDNA levels were measured using the StepOnePlus real time PCR System with primer sequences detecting the human mitochondrial NADH dehydrogenase 1 gene: forward CAAAGGCCCCAACGTTGTAG, and reverse CGGGTTTTAGGGGCTCTTTG as previously described^25–27^. Briefly, human nicotinamide adenine dinucleotide (reduced) (NADH) dehydrogenase 1 (MTND1) cDNA clone (SC101172, OriGene Technologies, Rockville, MD, USA) was used as a standard for mtDNA copy number. Equivalents of 10^9^, 10^8^, 10^7^, 10^6^, 10^5^, 10^4^, 10^3^, 10^2^, 10^1^, and 10^0^ copies of a plasmid encoding the MTND1 cDNA were amplified by real-time PCR and a standard curve was generated by plotting the copy numbers against the cycles, as previously described^28^. Details of the PCR protocol are available upon reasonable request. The cycle numbers obtained at a defined threshold from each sample were converted to total copy number by using the formula derived from the standard curve. The copy number /cell was calculated as follows: C = Q x V_DNA_/V_PCR_ × 1/No_EXT_, where C is the copy number/cell, Q is the quantity (total copy number) of DNA determined by the sequence detector in the PCR, V_DNA_ is the volume of cell DNA obtained after extraction (200 µl), V_PCR_ is the volume of cell DNA solution used for PCR (1 µl), No_EXT_ is the number of cells used for DNA extraction (5 × 10^6^). The copy number/µl plasma was calculated according to the following formula: c = Q x V_DNA_/V_PCR_ × 1/V_EXT_, where c is the concentration of mtDNA in plasma (copies/µl); Q is the quantity (total copy number) of DNA determined by PCR, V_DNA_ is the volume of plasma DNA obtained after extraction (200 µl), V_PCR_ is the volume of plasma DNA solution used for PCR (1µl), V_EXT_ is the volume of plasma used for extraction.

### Inflammasome stimulation in THP-1 cells and THP1-ASC-GFP cells

THP-1 cells were grown in RPMI-1640 (ThermoFisher Scientific, Gibco), supplemented with 10% FBS (ThermoFisher Scientific, Gibco) and 1% Penicillin/Streptomycin at 37 °C, 5% CO_2_ in a humidified atmosphere. For differentiation, cells were treated with 20 nM phorbol12-myristate-13-acetate (PMA) for 48 h before the experiments. On the day of the experiment, cells were shifted to RPMI-1640 medium without phenol red and primed with LPS (100 ng/ml) or left unprimed. For stimulation, 1 µg/ml poly(dA:dT)/LyoVec or 50 ng/ml nuclear PBMC-DNA or vehicle was immediately added to the medium for 1h, 6h and 24 h, respectively. After the experiment, tissue culture supernatants were collected and kept at -80°C until further analysis. For analysis of ASC specks, THP-1-ASC-GFP cells were grown in RPMI-1640 supplemented with 10% FBS, Normocin (100 µg/ml), Penicillin/Streptomycin (1%) at 37 °C, 5% CO_2_ in a humidified atmosphere. 2 × 10^4^ cells/well were seeded in 96-well plates and treated with PMA for 48 h before the experiments. For induction of ASC specks, cells were primed with LPS (200 ng/ml) or left unprimed for 2 h, before 50 ng/ml nuclear PBMC-DNA or 1 µg/ml poly(dA:dT)/LyoVec was added to the medium for 24h. PBMC-DNAs derived from three different AAA patients were used for stimulation and all stimulations were performed in triplicates.

### Image quantification and analysis of ASC specks

Images were obtained from a Keyence fluorescence microscope. 8 to 10 micrographs were taken per well, representing different image capture levels of the same image section. All micrographs were taken with the same exposure time at the same illumination intensity. The digital images were then used for counting the cell number by temporary setting the illumination intensity of the digital images to full brightness. This makes it possible to detect even faint cells with little GFP. In a second step, the illumination was reduced to count the ASC specks, recognizable by very bright condensed dots. An example is shown in the supplementary data (supplemental figure 6). Both, cell numbers and ASC specks were counted manually and the number of specks was normalized to the number of cells in the same picture. For statistics, three images per well were analysed by two-way ANOVA with adjusted *P*-values (Tukey’s multiple comparison test).

### Isolation of total RNA and protein lysates from cells

For extraction of total RNA, cells were washed in 1 × PBS and detached with RLT buffer (Qiagen RNeasy mini Kit). RNA was isolated by using RNeasy Mini Kit (Qiagen) following the instructions of the manufacturer and stored at − 80 °C until further use. Flow-through from RNA isolation was collected, and protein was precipitated in 4 Vol. acetone at − 20 °C, as described in the RNeasy Mini Kit manual. Pellets were resuspended in radio-immunoprecipitation assay (RIPA) buffer, supplemented with Phenylmethanesulfonyl Fluoride (PMSF) and stored at − 80 °C until further use.

### Quantitation of mRNA expression by real-time RT-qPCR

Real-time RT-PCR was performed as previously described^18^. Briefly, cDNA synthesis, 0.5 μg of total RNA was reverse transcribed using oligo-dT primers and SuperScript III reverse transcriptase following the manufacturer’s instructions. For real-time PCR, PowerSYBR Green master mix was added to appropriate cDNA samples and primers (Supplemental Tables 3 and 5). Samples were loaded onto 96-well PCR plates and analysed in a StepOnePlus real time PCR System. Quantitative analysis of gene expression was performed relative to expression of GAPDH and ACTB mRNA in corresponding samples by using the 2^−DDCt^ method^29^.

### Quantitation of IL-1β in the supernatant and from plasma

Supernatants and patient plasma were stored in aliquots at − 80 °C. Quantitation of IL-1β from supernatants and patient plasma was performed with Human IL-1β Mini TMB ELISA Development Kit and TMB ELISA Buffer Kit (PeproTech) following the instructions of the manufacturer.

### Immunoblotting

Protein concentrations in lysates were determined using the Pierce BCA Protein Assay Kit and 20µg of protein was loaded per lane of a Mini-Protean TGX gel (BioRad, 4–20%). Proteins were separated by electrophoresis using a Mini-Protean chamber (BioRad) with 1 × Tris/Glycine/SDS Running Buffer (BioRad). After separation, proteins were blotted on nitrocellulose in a Mini Protean chamber (BioRad) using Tris/Glycine Transfer Buffer. For detection, primary and secondary antibodies were used at concentrations as recommended by the manufacturers (Supplemental Table 3 and 4). Proteins were visualised with Super Signal West Dura ECL (ThermoFisher Scientific) using an Imaging System (Vilber Fusion).

### Statistical analysis

No statistical method was used to predetermine the sample size of the diagnostic study, but it was similar to those reported in previous studies on plasma biomarkers^30,31^. Microsoft Excel was used for data processing. GraphPad Prism 9 software was used for plot preparation, descriptive statistics and for statistical analysis. Data distribution was tested for normality by using the Kolmogorov–Smirnov Test and appropriate parametric or non-parametric tests were used. The specific tests used and *P*-value ranges are indicated in the figure legends. Univariate and multivariate analyses were performed using a logistic regression model. *P* values for regression coefficients were calculated by Wald tests. Variables that showed statistical significance in the univariate analysis were included in the multivariate analysis. The odds ratio (OR) was reported with a 95% CI. For the analysis of in vitro data, data are presented as mean +—SD and the number of technical or biological replicates in independent experiments is indicated in the figure legends.

## Results

### Plasma levels of ssDNA, dsDNA, and mtDNA are differentially distributed among AAA-patients, non-AAA vascular patients (controls) and healthy subjects

After additional review of the clinical data, plasma samples of 93 AAA patients and 89 vascular patients without AAA were eligible and included in an exploratory study design. Samples were matched for age, sex, BMI, and smoker status of the study participants (Table 1).

The AAA patients and controls showed a comparable frequency of hypertension, hyperlipidaemia and cardiovascular co-morbidities as well as a similar profile of routine blood parameters (Tables 1 and 2).

**Table 2:** Patient and control blood parameters.

Characteristic	AAA (N = 93) Median (IQR)	Non-AAA (N = 89) Median (IQR)	*P*-value
White blood cells (cells/nl)	7.61 (2.245)	7.69 (2.980)	0.9682
Erythrocytes (cells/nl)	4.6 (0.75)	4.4 (0.85)	0.0021
CRP (mg/L)	5.5 (7.205)	6.6 (6)	0.3591
Triglycerides (mg/dl)	118 (66.5)	124 (110)	0.6742
Total Cholesterol (mg/dl)	151 (64)	158 (58.5)	0.1615
LDL-Cholesterol (mg/dl)	87 (58.5)*	84 (38)*	0.6843
HDL-Cholesterol (mg/dl)	40 (18.5)*	41 (15)*	0.8511
Creatinine (mg/dl)	0.98 (0.365)	1 (0.485)	0.9093
eGFR (ml/min/1,73m^2^)	76.20 (27.85)	70 (36)	0.6450
Blood urea (mg/dl)	33 (11)	37 (24.5)	0.1715

CRP: C-reactive protein; LDL: low-density lipoprotein; HDL: high-density lipoprotein; eGFR: estimated glomerular filtration rate, *: data only available for a subset of patients (N = 29 AAA and n = 23 non-AAA); Statistics: Mann Whitney tests were used to test for the difference of medians.

In addition, an unmatched group of apparently healthy individuals without known CVD or AAA was used for comparison (Supplemental table 7). The maximum aortic diameter of AAA patients ranged between 35 and 102 mm (median 57 mm). In line with previous studies, a higher prevalence of peripheral artery disease (PAD), chronic obstructive pulmonary disease (COPD) and AAA family history was found in AAA patients compared with non-AAA patients, along with a lower prevalence of diabetes and Metformin therapy (Table 1). None of these diseases were known in the healthy control subjects and no information was available in this group about BMI, smoker status, hypertension, hyperlipidaemia, cardiovascular co-morbidities or blood parameters.

Plasma samples of all three groups were assessed for circulating mtDNA, ssDNA and dsDNA. They were compared to the content of intracellular mtDNA, ssDNA and dsDNA in corresponding PBMC, derived from the same blood sample (summarized in Table 3).

**Table 3:** DNA Concentrations in Plasma and PBMC.

	AAA N = 93 Median (IQR)	Non-AAA N = 89 Median (IQR)	Healthy N = 10 Median (IQR)	*P*-value AAA vs non-AAA	*P*-value AAA vs Healthy	*P*-value Healthy vs non-AAA
ssDNA (ng/ml Plasma)	298.5 (469.7)	142.6 (232.7)	122.8 (179.7)	< 0.0001	0.0012	0.6253
dsDNA (ng/ml Plasma)	54.0 (90.2)	19.0 (063.4)	30.2 (15.0)	< 0.0001	0.0205	0.1499
mtDNA (copies/µl Plasma)	3254 (11565)	713 (7306)	98.9 (291.2)	0.0063	0.0002	0.0110

	AAA N = 93 Median (Range)	Non-AAA N = 84 Median (Range)	Healthy N = 10 Median (Range)	*P*-value AAA vs non-AAA	*P*-value AAA vs Healthy	*P*-value Healthy vs non-AAA
ssDNA (ng/10^6^ cells)	6131 (4447)	5771 (4655)	6522 (1862)	0.5509	0.4578	0.3410
dsDNA (ng/10^6^ cells)	1499 (911)	1380 (851)	1542 (284)	0.1508	0.9338	0.5739
mtDNA (copies/ cell)	98.11 (80.2)	68.32 (54.07)	53.82 (38.08)	0.0020	0.0152	0.5245

AAA: abdominal aortic aneurysm; IQR: interquartile range, PBMC: peripheral mononuclear cells; The Kolmogorov Smirnov test was used to test for normality and lognormality. Mann Whitney tests were used to test for the difference of medians.

**Fig. 1:**
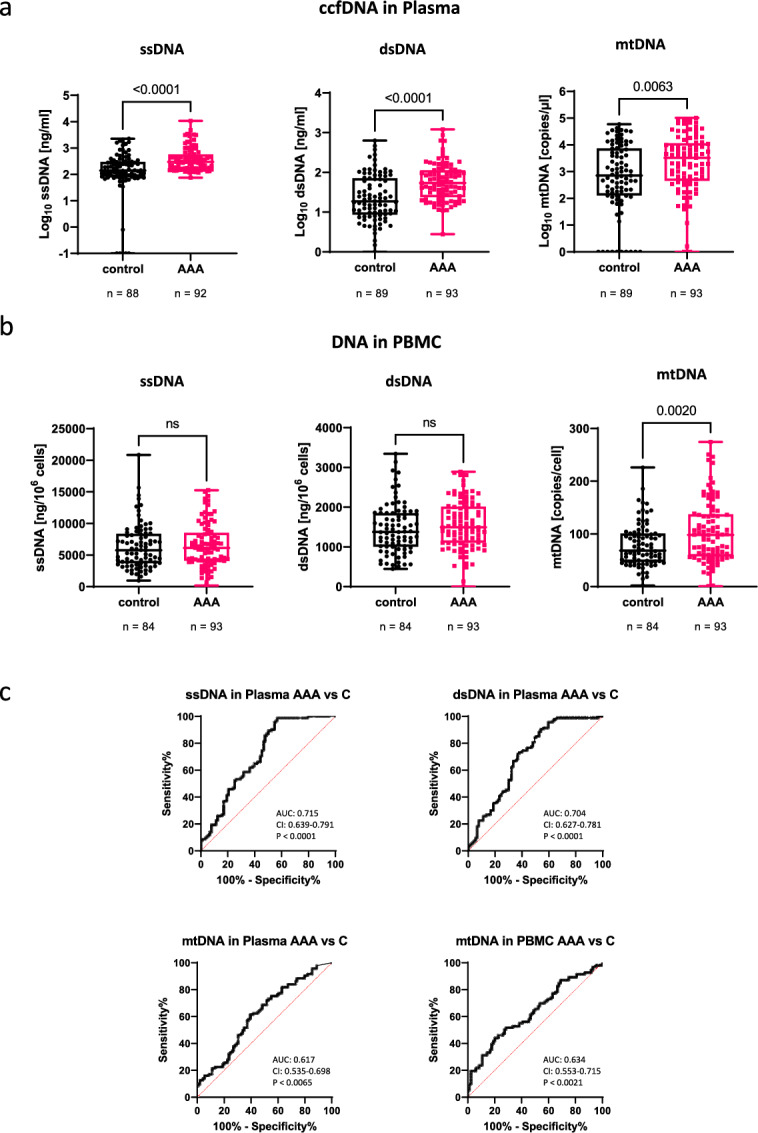
Comparison of DNA levels in plasma and in PBMC from AAA patients and controls. DNA was isolated from plasma, processed and quantified as described in the Materials and Methods section. (**A**) Levels of ssDNA, dsDNA and mtDNA in the plasma from AAA patients (red) and control patients (black). For dsDNA and mtDNA, n = 89 control samples and n = 93 AAA samples were used for analysis; For ssDNA, n = 88 control and 92 AAA samples were used for analysis. One sample in each group was excluded, here because of obvious measurement error. Statistics: Mann Whitney test with two-tailed *P*-value; each box plot shows all values as well as the upper quartile, the median, the lower quartile, minimum and maximum. (**B**) Copy numbers of mtDNA/cell are increased in PBMC from AAA patients. Note that the y-axes in A are depicted as a logarithmic scale. Statistics: Mann Whitney test with two-tailed *P*-value; each box plot shows all values as well as the upper quartile, the median, the lower quartile, minimum and maximum n = 93 AAA samples and n = 84 control samples. (**C**) Receiver operator characteristic (ROC) curves of ssDNA, dsDNA, or mtDNA in plasma and of mtDNA copies in PBMC, of the AAA group vs the control group (non-AAA), without adjustment for co-morbidities. AUC: Area under the curve. CI: confidence interval. *P*: *P* value. Data were plotted using GraphpadPrism software 9.0.0.

Each of the cfDNAs was significantly increased in patient vs control plasma samples (median ssDNA: 299 vs 143 ng/ml, *P* < 0.0001; median dsDNA: 54 vs 19 ng/ml, *P* < 0.0001; median mtDNA: 3254 vs 713 copies per µl, *P* = 0.063), (Fig. 1A). In addition, the copy number of mtDNA was significantly increased in PBMC derived from AAA patients vs control PBMC (median: 98,11 vs 68,32 copies/cell, *P* = 0.0020), whereas there was no difference in the content of ssDNA and dsDNA in PBMC from both groups, as expected (Fig. 1B, Table 3).

**Fig. 2:**
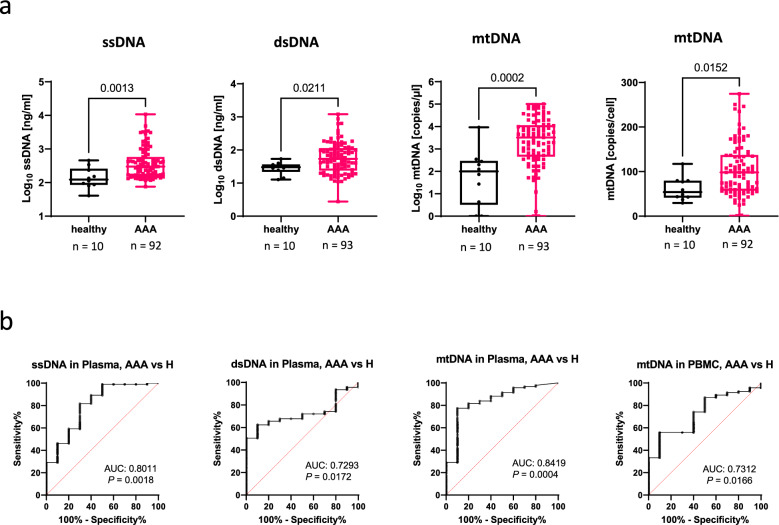
Comparison of DNA levels in plasma and in PBMC from AAA patients and healthy subjects. DNA was isolated from plasma, processed and quantified as described in the Materials and Methods section. (**A**) Levels of ssDNA, dsDNA and mtDNA in plasma as well as copy numbers of mtDNA/cell in PBMC of AAA patients (red) and controls (black). For analysis of the dsDNA levels in plasma, mtDNA copies in plasma and mtDNA copies in PBMC, n = 93 AAA samples and n = 10 control samples were included. For ssDNA, n = 10 control and 92 AAA samples were used for analysis. One sample was excluded from each group because of obvious measurement error. Statistics: Mann Whitney test with two-tailed *P* value; each box plot shows all values as well as the upper quartile, the median, the lower quartile, minimum and maximum (**B**). Receiver operator characteristic (ROC) curves of ssDNA, dsDNA, or mtDNA in plasma and of mtDNA copies in PBMC, of the AAA group vs the healthy subject group, without adjustment for co-morbidities. AUC: Area under the curve. CI: confidence interval. *P*: *P* value. Data were plotted using GraphpadPrism software 9.0.0.

The differences in plasma cfDNA and in the mtDNA copy number in PBMC were even higher between AAA patients and healthy subjects (median cf ssDNA: 299 vs 123 ng/ml, *P* = 0.0012; median cf dsDNA: 54 vs 30 ng/ml, *P* = 0.021; median cf mtDNA: 3254 vs 98,9 copies per µl, *P* = 0.0002; median mtDNA copy number: 98,11 vs 53,82 copies/cell), *P* = 0.015 (Fig. 2A, Table 3).

The cell-free ssDNA and dsDNA levels were well correlated, suggesting a common origin, whereas mtDNA did not correlate with the levels of ssDNA or dsDNA in plasma samples, suggesting a different origin or, alternatively a different stability of mtDNA in plasma (Supplemental figure S1A). The cellular levels of mtDNA, ssDNA and dsDNA were well correlated in PBMC, as expected (Supplemental figure S1B). Spearman correlation analysis of the clinical data revealed a weak negative correlation between dsDNA plasma levels and the amount of total cholesterol, a positive correlation between ssDNA plasma levels and the amount of total cholesterol and with the maximal AAA diameter of the patients, as well as a weak positive correlation between the mtDNA copy number in plasma and urea. The correlation analyses of the cfDNAs with age, BMI, maximal AAA diameter, white blood cell numbers, C-reactive protein, cholesterol, creatinine and urea levels in the plasma of patients are summarized in supplementary table 8. It should be noted, however that some of the clinical parameters were only available from a subset of patients.

**Fig. 3:**
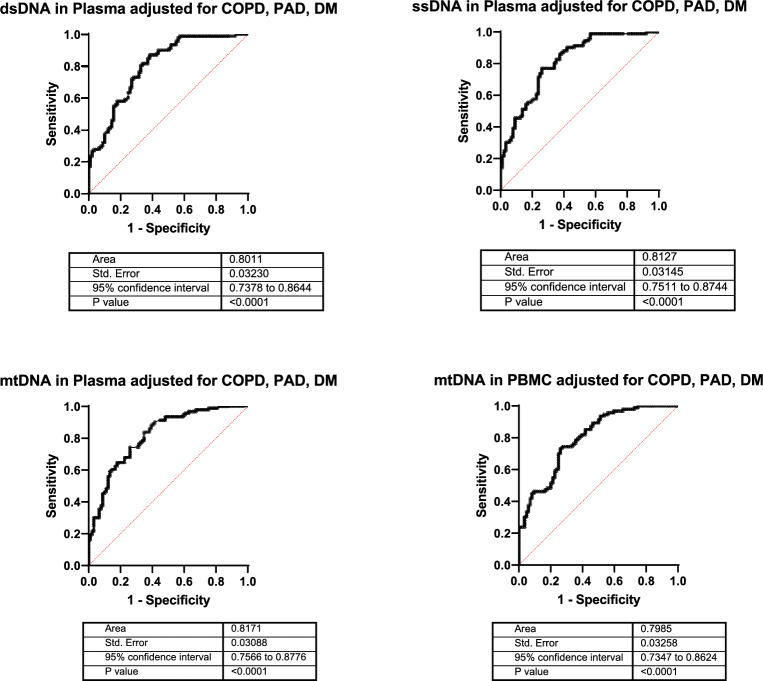
Receiver operator characteristic (ROC) curves of ssDNA, dsDNA, or mtDNA in plasma and of mtDNA copies in PBMC, of the AAA group vs the control group (non-AAA), after adjustment for co-morbidities. Data were plotted using GraphpadPrism software 9.0.0. AUC: Area under the curve. CI: confidence interval. *P*: *P* value. COPD: chronic obstructive pulmonary disease, PAD: peripheral artery disease, DM: Diabetes mellitus.

The diagnostic marker potential of the two nuclear cfDNAs in plasma was similar with a significant diagnostic power for AAA patients, vs controls, showing an AUC value of 0.715, *P* < 0.0001 for ssDNA, and an AUC value of 0.704, *P* < 0.0001 for dsDNA. In contrast, the diagnostic marker potential of mtDNA was low with an AUC value of 0.617, *P* = 0.0065 for cell-free mtDNA in plasma, and an AUC value of 0.634, *P* = 0.0021 for the mtDNA copy number/cell (Fig. 1C). The diagnostic marker potential of the three cfDNAs in plasma was much better for differentiating AAA patients from healthy subjects, showing an AUC value of 0.801, *P* = 0.0018 for ssDNA, an AUC value of 0.729, *P* = 0.0172 for dsDNA, and an AUC value of 0.842, *P* = 0.0004 for mtDNA. The diagnostic marker potential of the mtDNA copy number/cell for AAA patients, vs healthy subjects displayed an AUC value of 0.731, *P* = 0.0166 (Fig. 2B). To test for potential confounders, we next conducted a univariable analysis by binary logistic regression for the investigated cfDNA plasma levels, the mtDNA copy number in PBMC as well as for patient co-morbidities, smoking status, age and sex (Supplemental table 9). Parameters with a P-value of less than 0.1 were then used in a multivariate analysis. After adjusting for comorbidities, the AUC was 0.801 for dsDNA, 0.813 for ssDNA, 0.817 for mtDNA in plasma, and 0.799 for mtDNA in PBMC (Fig. 3).

The positive and negative predictive power was 76.1% and 69.72% for dsDNA, 77.46% and 69.72% for ssDNA, 78.38 and 71.30% for mtDNA in plasma, and 75.0% and 69.72% for mtDNA in PBMC. The amount of dsDNA, and the amount of ssDNA, proved to be independent predictors of AAA presence, along with COPD (Table 4). In addition, the mtDNA copy numbers in plasma and PBMC displayed significant values, however with odds ratios of 1.00, indicating that they are equally likely in both groups. (Supplemental tables 10 and 11).

**Table 4:** Multivariate logistic regression of AAA presence including the amount of ssDNA and dsDNA in plasma and co-morbidities.

Parameter	Sig. different than zero *P*-value	Odds ratio	95% CI lower value	95% CI upper value
**Amount of ssDNA in Plasma**	0.0220	2.065	1.181	4.097
PAD	< 0.0001	0.1014	0.0416	0.2248
COPD	0.0112	5.769	1.626	25.42
Diabetes mellitus	0.0279	0.4037	0.1763	0.8959
**Amount of dsDNA in Plasma**	0.0482	28.87	1.442	1087
PAD	< 0.0001	0.096	0.04	0.21
COPD	0.01	6.069	1.673	27.13
Diabetes mellitus	0.0363	0.4337	0.1953	0.940

CI, confidence interval; COPD, chronic obstructive pulmonary disease; DNA, deoxyribonucleic acid; ds, double strand; GraphPadPrism 9.0.0 software was used to calculate odds ratios, CI and *P* values. *P* values for regression coefficients were calculated by Wald tests.

### Purified total DNA from patients with AAA stimulates an AIM2 inflammasome response in THP-1 macrophages

Because dsDNA in plasma appeared to be the most promising candidate as a biomarker in our analysis, we next aimed to investigate its functional role in AAA. Based on our previous studies, where we had detected increased AIM2 inflammasome levels in AAA-tissue and in PBMC from AAA patients^18,21,22^, as well as an induction of AIM2 expression in vascular smooth muscle cells in response to cell debris^20^, we assumed that cell-free dsDNA might be able to induce an AIM2 inflammasome response in inflammatory cells. To test this hypothesis, we challenged PMA-differentiated THP-1 macrophages with isolated DNA derived from blood cells of different patients. THP-1 cells are commonly used as a model for inflammasome activation, because they endogenously express AIM2, ASC, NLRP3 and Pro-Caspase-1^32^. In a first step, we compared the induction of *AIM2* and *IL1B* transcripts in LPS primed THP-1 cells, stimulated with PBMC-DNA derived from four healthy donors, three non-AAA donors and three AAA donors. Interestingly, induction of *AIM2* expression was weaker in response to DNA from healthy individuals than to DNA from AAA and non-AAA patients after six hours. The effect was equalized after 24 h. No difference was observed for *IL1B* expression. (Supplemental figure S2). Because cell-free DNA is often combined with proteins, such as histones, nucleosomes and DNA-binding proteins, we next compared induction of a set of inflammasome genes in LPS-primed THP-1 cells upon additional stimulation with Chromatin extracts derived from white blood cells of non-AAA and AAA patients (Supplemental methods). Compared with LPS priming alone, the combined stimulation with chromatin from non-AAA or AAA patients resulted in reduced *AIM2* but increased *IL1B* gene expression (Supplemental Figure S3A). Moreover, chromatin stimulation resulted in a weaker induction or even repression of eight inflammasome genes except *Il1B,* when compared with pure PBMC-DNA, indicating different responses of these genes to different DNA types (Supplemental Figure S3B). Based on these preliminary experiments, we decided to study the response to pure PBMC-DNA derived from AAA patients in more detail.

**Fig. 4:**
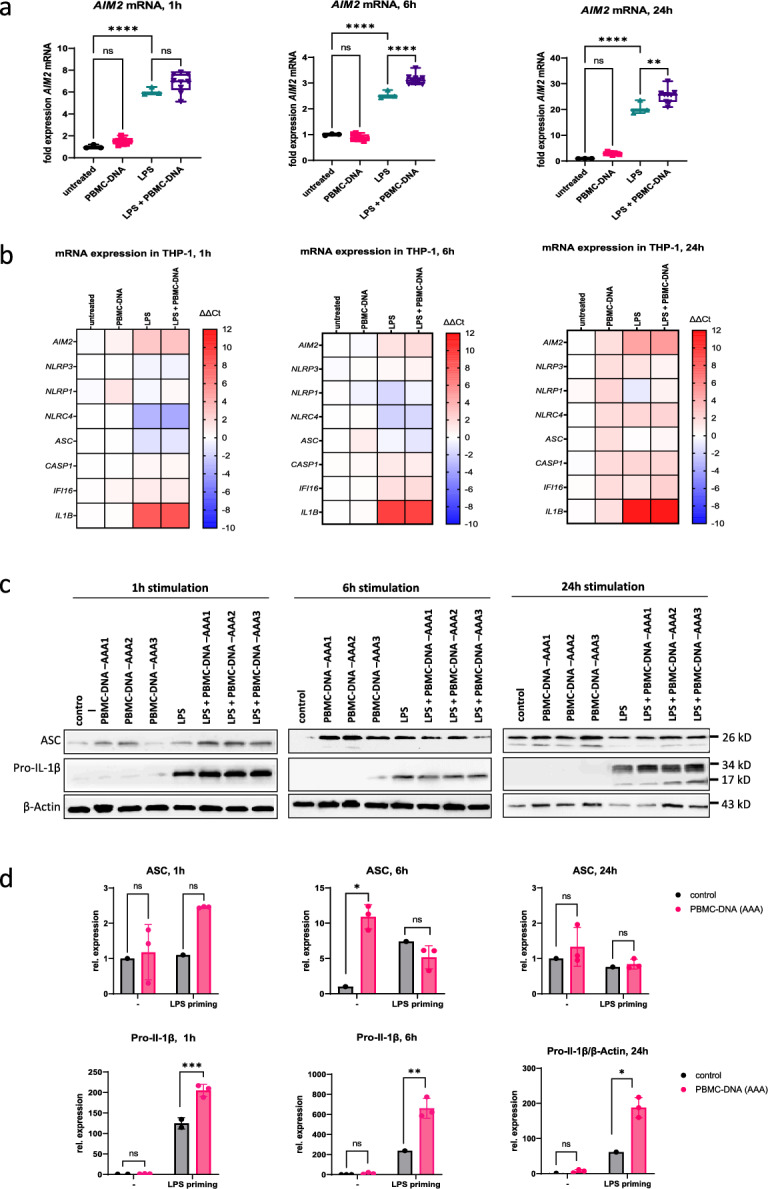
Inflammasome activation by PBMC-DNA in THP-1 cells. THP-1 cells were differentiated with 20 nM phorbol-12-myristate-13-acetate (PMA) for two days and subsequently stimulated with vehicle, or 50 ng/ml PBMC-DNA from AAA-patients (n = 3), in the presence or absence of 100 ng/ml LPS (for 6h or 24h as indicated). For 1h stimulation, PMA-differentiated cells were left untreated or primed for 2h with 200 ng/ml LPS before stimulation (**A**) Relative expression of *AIM2* mRNA (fold change vs. untreated), as determined by RT-qPCR. Data are shown as box plots of n = 3 (untreated, LPS treated) to 9 (PBMC-DNA, LPS + PBMC-DNA) ΔΔCT values. Statistics: Ordinary one-way ANOVA with adjusted *P*-values (Šídák’s multiple comparison test) (**B**) Relative expression of inflammasome genes in PMA-differentiated THP-1 after stimulation with vehicle, or 50 ng/ml PBMC-DNA from AAA-patients (n = 3), in the presence or absence of LPS. Data were normalized to untreated control and means are shown as a heatmap. n = 3 (untreated, LPS treated) to 9 (PBMC-DNA, LPS + PBMC-DNA). ΔΔCT values were used for calculation of reach relative expression. (**C**) Representative immunoblot analysis of ASC, Pro-Caspase-1 and Pro-IL1β in THP-1 cell lysates after stimulation. (**D**) Normalized amount of ASC and Pro-Il-1β protein expression derived from immunoblotting. Red bars show the means of three PBMC-DNAs. Data were normalized to the expression of β-Actin and shown as fold expression of control. Statistics: Two-way ANOVA with adjusted *p*-values (Šídák’s multiple comparison test). ns: not significant, * *P* < 0.05, ** *P* < 0.01, *** *P* < 0.001, **** *P* < 0.0001.

Real-time RT-PCR revealed a time-dependent, differential induction of eight inflammasome genes upon stimulation with PBMC-DNA (Fig. 4A, B and supplemental figure S4). Compared to LPS-primed THP-1 cells alone, *AIM2* mRNA expression was significantly increased by additional stimulation with PBMC-DNA after 6 and 24 h, with a trend to increase already after 1 h (Fig. 4A). In addition, *IFI16*, encoding another DNA sensor protein, was significantly induced by PBMC-DNA in LPS-primed and unprimed THP-1 cells after 24 h (Fig. 4B and supplemental figure S4). *IL1B* mRNA expression was most strongly increased by LPS priming, with further enhancement by PBMC-DNA, 1 h after stimulation. After 6 and 24 h, *IL1B* mRNA expression was further increased in LPS primed THP-1 cells, but without displaying an additional effect by PBMC-DNA (Fig. 4B and supplemental figure S4). All inflammasome genes, except for *IL1B* were significantly induced in unprimed THP-1 cells by PBMC-DNA after 24 h (Fig. 4B and supplemental figure S4).

Immunoblot analysis of inflammasome proteins revealed an increased amount of ASC protein already after 1 h of stimulation with PBMC-DNA (Fig. 4C, D), although the ASC mRNA amount was decreased at this time point. The increase was even stronger in LPS-primed THP-1 cells. No difference in ASC protein levels was detectable after 24 h (Fig. 4C, D). Pro-Il-1β was significantly increased after 1, 6 and 24 h of stimulation with PBMC-DNA in LPS-primed THP-1 cells compared to LPS-primed THP-1 cells alone (Fig. 4C and D). The amount of the sensor proteins NLRP3, NLRC4 and AIM2 was too low to be detected by immunoblotting (Supplemental figure S5).

**Fig. 5:**
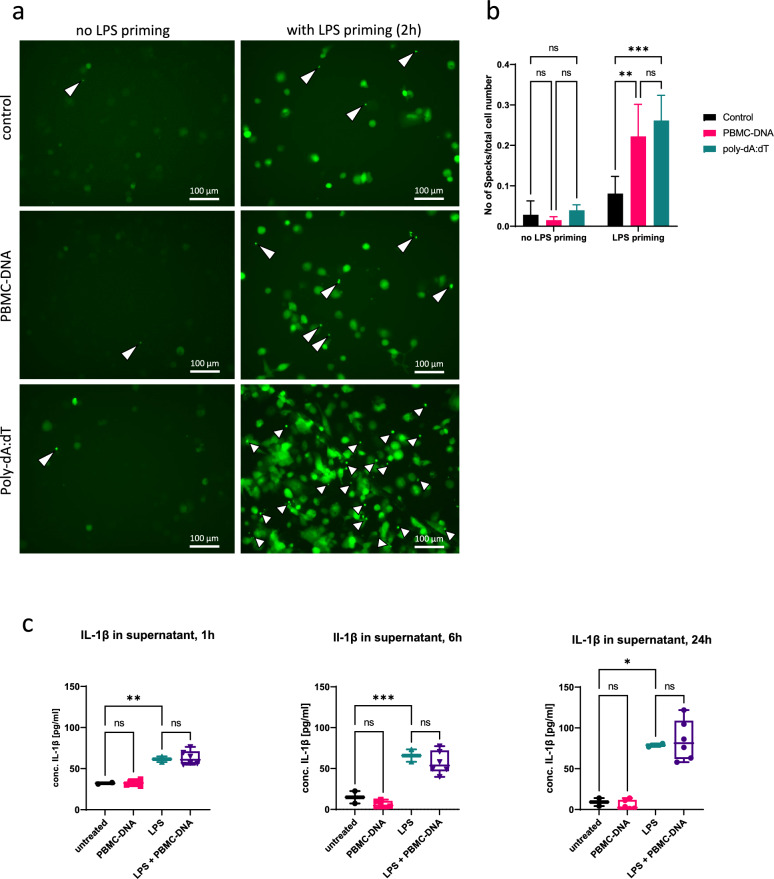
Inflammasome activation by PBMC-DNA in THP-1-ASC-GFP cells (**A**) Representative fluorescence micrographs of ASC specks induced by PBMC-DNA in THP-1-ASC-GFP. THP-1-ASC-GFP cells were differentiated with 20 nM phorbol-12-myristate-13-acetate (PMA) for two days, primed for 2h with 200 ng/ml LPS or vehicle, and stimulated for 24h with vehicle, or 50 ng/ml PBMC-DNA from AAA-patients (n = 3). White arrows point to ASC specks, representing inflammasomes. Images were taken with a Keyence microscope at an original magnification of 200x. (**B**) Quantification of ASC specks per cell number (n = 3 to 9 images per treatment). Statistics: Two-way ANOVA with adjusted *P*-values (Tukey’s multiple comparison test) (**C**) Release of IL-1β into the supernatant of THP-1 cells, derived from the experiments in Fig. 1. Concentration of IL-1β was determined by ELISA. Data are derived from three biological replicates analysed in triplicate. Statistical analysis was performed by ordinary one-way ANOVA with subsequent Šídák’s multiple comparison test (1h and 6h) or by mixed-effect analysis (24h). ns: not significant, * *P* < 0.05, ** *P* < 0.01, *** *P* < 0.001.

To evaluate the assembly of inflammasome protein complexes in response to PBMC-DNA, we used fluorescent ASC reporter cells for visualization of ASC speck formation (Fig. 5A). In untreated THP1-ASC-GFP cells, fluorescent ASC was hardly visible, representing its soluble and diffuse cytoplasmic and nuclear distribution. Priming with LPS resulted in diffuse enhanced green fluorescence, representing ASC induction and polymerization. Upon stimulation with PBMC-DNA or poly-(dA:dT), ASC speck formation was induced in both LPS-primed and unprimed cells, representing mature inflammasome protein complexes. The formation of ASC specks was already visible one hour after LPS-priming/PBMC-DNA stimulation on a low level (examples are shown in supplementary figure S6A) and was fully developed after 24 h. The number of ASC specks per imaging field was significantly increased by PBMC-DNA in LPS-primed THP1-ASC-GFP cells compared with cells only primed with LPS or untreated cells (Fig. 5B).

Despite the strong increase in expression of inflammasome genes and induction of ASC specks, the release of IL-1β from stimulated THP-1 cells was not further increased by PBMC-DNA compared with untreated or LPS-primed THP-1 cells, respectively, neither after 1, 6, or 24 h. (Fig. 5C). In contrast, intracellular activation of caspase-1 was further enhanced in LPS-primed, PBMC-DNA-stimulated cells compared to cells primed with LPS alone (Supplementary figure S7A).

### Dose–response to PBMC-DNA samples containing high mtDNA levels

**Fig. 6:**
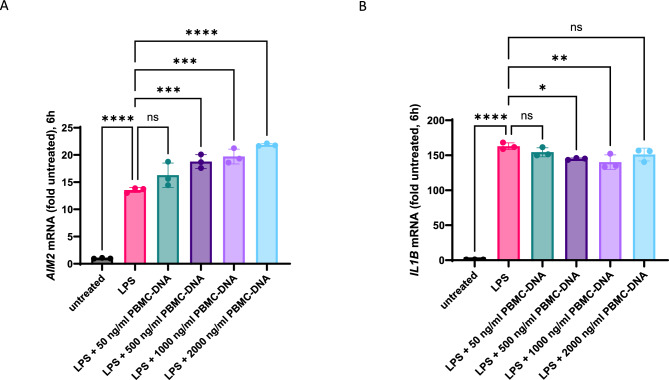
*AIM2* expression in response to increasing doses of PBMC-DNA containing high mtDNA levels (mtDNA^high^-mix). Expression of *AIM2* mRNA (**A**) and *IL1B* mRNA (**B**) was determined by RT-qPCR using specific primers. A strong induction of *AIM2* mRNA was observed after 1h priming with 100 ng/ml LPS. After additional stimulation for 5 h with increasing doses of the mtDNA^high^-mix, *AIM2* mRNA expression was further increased. Statistical analysis was performed by ordinary one-way ANOVA with subsequent Šídák’s multiple comparison test. ns: not significant, * *P* < 0.05, ** *P* < 0.01, *** *P* < 0.001, **** *P* < 0.0001.

To investigate, whether the higher cell-free mtDNA content in PBMC-DNA derived from AAA patients might be responsible for the inflammatory response, we selected four patient samples from our cohort, with a particularly high mtDNA copy number in their PBMC and high levels of mtDNA in their corresponding plasma samples to use as a mix for stimulation (mtDNA^high^-mix). PMA-differentiated and LPS-primed THP-1 cells were stimulated with increasing doses of this mtDNA^high^-mix for six hours. Compared to LPS-primed THP-1 cells alone, *AIM2* mRNA expression was significantly increased by additional stimulation with the PBMC-DNA mix in a dose responsive manner, whereas *IL1B* mRNA expression remained unchanged or even reduced (Fig. 6).

### IL-1β levels are slightly elevated in the plasma of AAA patients

As a readout for inflammasome activation in vivo, we finally measured IL-1β levels in patient plasma. Only 21/89 control samples in contrast to 42/93 AAA samples were positive for IL-1β (*P* = 0.003, Fisher’s exact test). Although values were moderately increased in AAA patients versus controls (Supplementary figure S7B), there was no correlation of IL-1β levels with dsDNA, ssDNA or mtDNA levels (Supplementary figure S7C).

## Discussion

Herein we report the diagnostic value of cell-free ssDNA, dsDNA and mtDNA in the plasma of AAA-patients and its potential to induce an AIM2-mediated inflammatory response in human THP-1 macrophages. Our data suggest that elevated cfDNA levels may contribute to chronic inflammation via AIM2 inflammasome activation in patients with AAA. In addition, we found that the mtDNA copy number is significantly higher in the PBMC of AAA patients than in PBMC of control patients. The contribution of the latter to AAA disease is unknown and can only be speculated at present. Possibly, its extracellular release into the circulation may have systemic effects via the innate immunity. There is evidence for increased mtDNA release caused by mutational mtDNA stress, and extracellular mtDNA release has been shown to increase circulating mtDNA, thereby inducing age-associated inflammation with systemic effects on many tissues^33^. Genetic alterations in the mitochondrial genome of AAA patients have recently been shown to be associated with AAA^34^. However, the exact mechanism of how the mutated mtDNA contributes to AAA has yet to be investigated and is beyond the scope of this study.

Over the past 20 years, numerous studies have investigated the suitability of circulating plasma parameters as potential biomarkers for AAA monitoring (for review see^35–37^). Only a few of these studies have focused on the role of cfDNA or DNA complexes. Elevated levels of total cfDNA in the plasma of AAA-patients have been described, however, without further analysis of its diagnostic value and consequences for maintaining the course of the disease^30,38,39^. A study on oxidized nuclear and mitochondrial DNA revealed increased amounts of non-oxidized and total nuclear DNA and a trend for increased levels of oxidized mitochondrial DNA in plasma samples of patients with AAA^38^. Other studies used DNA-histone complexes and cfDNA as neutrophil activation markers in the plasma of AAA patients^30,39^ or in animal models^40^, without giving further detailed analysis on the composition and distribution of different cfDNA types. To our knowledge, there is only one study analysing the plasma levels of dsDNA, ssDNA and mtDNA separately, namely in patients with type 2 diabetes^26^. The authors found that particularly mtDNA was increased in diabetic patients compared with healthy subjects and that the mtDNA from these patients could induce AIM2 inflammasome-dependent caspase-1 activation and IL-1β secretion in murine LPS-primed bone marrow derived macrophages (BMDM).

The results of quantitative studies using cell-free DNA in plasma rely on the analytical performance of the assay used for quantification. In addition, pre-analytic parameters of blood collection time and delayed DNA preparation from the samples may affect the results obtained^41,42^. This study benefits from a clinically optimized analytical process used by the VBBH (blood sampling at the time of patient admission to the hospital, i.e. before treatment; plasma preparation within 1–4 h after venepuncture; additional centrifugation step to eliminate remaining cells or platelets in the plasma sample; immediate storage at − 80 °C; only one time thawing and immediate DNA extraction (see Methods section)). Our findings suggest that fluorometrically quantified dsDNA has the highest diagnostic value for AAA among the three tested cfDNA types, according to multiple logistic regression analysis. The specificity of the cell-free dsDNA may not be sufficient to be considered as a single marker for AAA. However, it might be combined in a marker panel with additional plasma markers such as D-Dimers, and/or neutrophil derived markers, such as citH3 or MPO, which are also increased in AAA^30,31,43^. In addition, the prognostic value of dsDNA as a biomarker for monitoring progression and rupture risk of AAA patients remains to be investigated in future studies. It should be mentioned here that the dsDNA that was quantified fluorometrically, probably contains both nucleic and mtDNA. Which of the two predominates cannot be determined from our measurements. According to other studies, mtDNA is the predominant nucleic acid in cell-free DNA, with up to 50000-fold more mitochondrial than nuclear copies in the plasma of healthy individuals and 3000- to 10000-fold more mtDNA copies in patients suffering from cancer or diabetes^26,41^.

In addition to differences in the level of cfDNA in plasma, we here detected a significantly increased mtDNA copy number in PBMC of AAA patients compared with control samples. Interestingly, this agrees with our recent finding showing increased mtDNA copy numbers in vascular smooth muscle cells (VSMC) derived from AAA patients (AAA-SCM) compared with healthy VSMC^25^. The mtDNA copy number has recently become a popular biomarker for mitochondrial function in aging and diseased tissues and may have a diagnostic value for a number of diseases and disease states^44^. However, it must be interpreted carefully for different reasons. First, the mtDNA copy number in tissue cells may be considered a proxy indicator for mitochondrial activity, the alteration of which reflects mitochondrial biogenesis and function. Second, the mtDNA copy number varies considerably between different cell types and tissue origin from less than 100/cell to several thousand (heart and skeletal muscle) or even 100 thousand in oocytes^44,45^. In PBMC, the average copy number ranges from ~ 150 in neutrophils to ~ 600 in B- and T-lymphocytes^46^. Accordingly, in white blood cells, it is a measure of hematological and hematopoiesis biology, reflecting the capacity to respond to physiological resources available during a disease state^46^. The increased mtDNA copy number detected in PBMC of AAA patients, here may thus be interpreted in two different ways. Either, it reflects increased mitochondrial biogenesis in all PBMC cell types as a response to the disease state or mutation within the mtDNA, or alternatively, it reflects a shift of the white blood cell composition to a higher proportion of lymphocytes and activated monocytes or platelets. Unfortunately, we cannot tell from our data which of the two mechanisms applies here, because no information on the differential blood count was available from the patients. The fact that the CVD risk was recently shown to be associated not with an increase but rather with a decrease in mtDNA copy number^47^ argues for the second explanation, namely a shift in the composition of white blood cells in AAA patients to more B-cells and platelets. The exact mechanism, how increased mtDNA copy numbers and its potentially increased extracellular release contribute to AAA pathology, remains to be investigated.

Chronic inflammation and activation of inflammasomes has been shown to be associated with AAA^48^. According to our previous studies, the AIM2 inflammasome, which detects foreign and the cell’s own nuclear and mitochondrial dsDNA in the cytoplasm, appears to be increased in both the aortic wall of AAA patients and in their PBMC^18,21–23^. Having identified dsDNA as an independent marker for AAA monitoring here, we additionally addressed its effect as a damage associated molecular pattern (DAMP) to induce and maintain an inflammasome response. Our results suggest that cell-free DNA-mediated AIM2 inflammasome activation is associated with chronic inflammation in patients with AAA. In particular, PBMC-DNA derived from AAA patients appears to increase the transcription of *AIM2* in THP-1 macrophages within 1–6 h, i.e. it acts at a very early stage of the innate immune response even in the absence of priming agents such as LPS. Within 24 h after addition to THP-1 macrophages, PBMC-DNA induced the transcription of the whole set of inflammasome genes, including *AIM2, IFI16, NLRP3, NLRP1, NLRC4, ASC, CASP1* and *IL1B*, indicating that the AIM2 inflammasome is not the only pathway activated in response to persistent presence of foreign DNA in unprimed THP-1 macrophages.

Our data agrees well with previous studies on cell response to misplaced DNA. AIM2 was the first dsDNA sensor in the cytosol described and the first inflammasome shown to recognize synthetic or pathogen-derived dsDNA in murine BMDM and in human THP-1 macrophage cells^49–52^. Later, other pathways were described to recognize dsDNA leaked from the nucleus or mitochondria (for review see^53^). IFI16 was shown to induce expression of IFN-β upon sensing dsDNA without inducing an inflammasome^54^. The cyclic GMP-AMP synthase (cGAS) was shown to sense misplaced genomic, mitochondrial, and microbial dsDNA and to mobilize stimulator of interferon genes (STING)^55^. The cGAS-STING pathway in turn may drive activation of NLRP3 and thereby result in inflammasome activation^56^. Finally, in human keratinocytes, dsDNA was recently demonstrated to be dependent on NLRP1 activation^57^. Taken together, the response to foreign or misplaced DNA appears to be complex and involves more than just a single pathway. However, only activation of *AIM2* and *NLRP3* is linked to inflammasome assembly, resulting in activation of caspase-1 and subsequent cleavage of Pro-Il-1β into its active form.

In this study, only *AIM2* and *IFI16* showed sustained increased transcription in response to PBMC-DNA in LPS-primed THP-1 macrophages even after 24 h. Together with our finding that PBMC-DNA could also increase ASC speck formation and caspase-1 activation in LPS-primed THP-1 cells, this argues for a major role of the AIM2 inflammasome, although the additional activation of other inflammasomes sensed by cGAS-STING-NLRP3 or NLRP1 cannot be excluded.

Regardless of the results in this study, some questions remain regarding their transferability to the situation in situ in the aortic wall. First, full induction of inflammasome activity by PBMC-DNA required LPS-priming of the THP-1 cells, whereas the effect was weak or absent in non-primed cells. This argues for a synergistic effect of the cfDNA and raises the question, of what could be a priming mechanism in AAA. There is recent evidence that bacterial derivatives contribute to the progression of AAA. Gut dysbiosis and bacterial translocation to the blood and aneurysmal wall has been demonstrated in patients with AAA^58^. Moreover, human AAA samples were shown to contain bacterial DNA with high frequency, and in particular that of Porphyromonas gingivalis, the most prevalent pathogen involved in chronic periodontitis. The authors further demonstrated that neutrophil activation in human AAA was associated with NET formation in the intraluminal thrombus, leading to the release of cfDNA^39^. Other studies demonstrated the presence of Helicobacter cinaedi particles in the aneurysm walls^59^. Although a final proof is pending, even if no living bacteria were present within the aneurysmal wall, their components and derivatives, including LPS, might be sufficient for priming local inflammatory cells and activate innate immunity which is further enhanced by cfDNA. The second question relates to the fact that DNA extracted from peripheral blood does not necessarily have to have the same composition as cfDNA in the aortic wall of an aneurysm. We here used purified DNA that might differ from the one released in aneurysmal tissues. Native cfDNA is often complexed with proteins, such as histones, nucleosomes, and DNA-binding proteins, providing stability and protection to the DNA fragments. This complexed DNA may differ in its composition between healthy individuals and patients and might induce other inflammatory responses than pure DNA. To address this question, we used chromatin derived from PBMC of different healthy, non-AAA and AAA-donors for stimulation of THP-1 cells. These preliminary experiments indeed revealed different inflammatory responses which, however, will need to be examined in more detail elsewhere.

The most interesting type of DNA in connection with the triggering of an immune response may be mtDNA, which was increased in both plasma and PBMC of AAA-patients in this study. It is well documented that mitochondrial DNA release triggers inflammation in cardiovascular and other diseases. Circulating cell-free mtDNA that was released passively due to physical damage or actively as a component of NETs can bind to pattern-recognition receptors (PRRs) on several cell types and act as an inducer of the innate immune system^33^. Accordingly, dsDNA from patients with type 2 diabetes, containing a high amount of mtDNA, has been shown to induce AIM2-dependent caspase-1 inflammasome activation and IL-1β release in macrophages^26^. The PBMC-derived dsDNA samples from AAA patients that were used for the THP-1 experiments here, were selected due to their high mtDNA content. In addition, these mtDNA^high^ samples showed a dose–response effect on AIM2 expression. Together this suggests that mtDNA in particular may be responsible for the induction of AIM2 expression and the inflammasome response.

In summary, our data are in line with the hypothesis that cell-free DNA, derived from AAA patients triggers a proinflammatory response in macrophages by activating the AIM2 inflammasome and thus could be a driving force for the chronic inflammation observed in these patients.

We are aware that our conclusions are based on the use of a cell culture model, whereas the actual situation in the aneurysmal aortic wall is probably even more complex and involves responses to different combinations of cfDNA and priming molecules.

## Supplementary Information

Supplementary Information 1.

Supplementary Information 2.

## Data Availability

All processed data generated or analysed during this study are included in this published article and its supplementary information files. Additional raw data from measurements are available upon reasonable request from the corresponding author. Clinical data are not openly available due to reasons of sensitivity and are available from the corresponding author in an anonymous form upon reasonable request. Data are located in controlled access data storage at the Universitätsklinikum Heidelberg.
