# Supplementary material for: Exploring circulating cell-free DNA as a biomarker and as an inducer of AIM2-inflammasome-mediated inflammation in patients with abdominal aortic aneurysm
Source: Sci Rep. 2025 Jun 20;15:20196. doi: 10.1038/s41598-025-06220-5 (PMC12181305; doi:10.1038/s41598-025-06220-5)
Supplement: Supplementary file 1 — Supplementary Information 1. [file 41598_2025_6220_MOESM1_ESM.pdf]

Supplemental materials to

Exploring circulating cell-free DNA as a biomarker and as an inducer of AIM2-inflammasome-mediated inflammation in patients with abdominal aortic aneurysm

Authors: Susanne Dihlmann[1*], Carolin Kaduk[1], Karola H. Passek[1], Anja Spieler[1], Dittmar Böckler[1] and Andreas S. Peters[1,2]

Supplemental table 1: commercial assay kits used throughout the study

Name	Source	Identifier
Caspase-Glo 1 Inflammasome Assay	Promega, Walldorf, Germany	G9951
DNeasy Blood and Tissue Kit	Qiagen, Hilden, Germany	69504
Human IL-1β Mini TMB ELISA Development Kit	PeproTech	900-TM95
QuantiFluor dsDNA System	Promega, Walldorf, Germany	E2670
QuantiFluor ssDNA System	Promega, Walldorf, Germany	E3190
Pierce BCA Protein Assay Kit	Life Technologies GmbH	23225
RNeasy Mini Kit	Qiagen, Hilden, Germany	74104
TMB ELISA Buffer Kit	PeproTech	900-T00

Supplemental table 2: plasmids and cell lines used throughout the study

Name	Source	Identifier
human nicotinamide adenine dinucleotide (reduced) (NADH) dehydrogenase 1 (MTND1) cDNA clone	OriGene Technologies, Rockville, MD, USA	SC101172
THP-1 cells	DSMZ-German Collection of Microorganisms and Cell Cultures GmbH, Leibnitz Institute	ACC-16
THP-1-ASC-GFP cells	InVivoGen, Europe	Thp-ascgfp

Supplemental table 3: reagents and cell culture media used throughout the study

Name	Source	Identifier
DNase I Amp grade	Life Technologies GmbH	18068015
dNTP Set 100 mM solutions	Life Technologies GmbH	R0182
Fetal Bovine Serum (FBS)	Life Technologies GmbH	10500064
Ficoll Paque Plus	Cytivia, Sigma (Merck)	17-1440-02
4 x Laemmli Sample Buffer	BioRad	1610747
Lipopolysaccharide (LPS; TLR4 agonist)	Sigma (Merck)	L3024
Mini-Protean TGX Gels, 4-20%	BioRad	4561093
oligo-dT 12-18 primers	Life Technologies GmbH	18418012
Penicillin/streptomycin	Gibco, ThermoFisher Scientific	15140122
Phenylmethanesulfonyl Fluoride (PMSF)	Cell Signaling Technology	8553
Phorbol-12-myristate-13-acetate (PMA)	Sigma (Merck)	P1585
Phosphate buffered saline (PBS)	Gibco, ThermoFisher Scientific	20012019
Poly(dA:dT)/LyoVec	Invivogen	tlrl-patc
PowerSYBR Green master mix	Life Technologies GmbH	4367659
Radio-Immunoprecipitation assay (RIPA) buffer	Cell Signaling Technology	9806
Recombinant RNasin RNase Inhibitor	Promega, Walldorf, Germany	N2511
RPMI-1640 Medium, with L-glutamine and sodium bicarbonate	Sigma (Merck)	R8758
RPMI-1640 w.o. phenol red	Gibco, ThermoFisher Scientific	11835030
SuperSignal West Dura ECL Reagent	ThermoFisher Scientific	37071
SuperScript III reverse transcriptase	Life Technologies GmbH	18080044
10 x Tris/Glycine/SDS Running Buffer	BioRad	161-0732

10 x Tris/Glycine Transfer Buffer	BioRad	161-0734
TrypLE Express Enzyme	Life Technologies GmbH	12604013

Supplemental table 4: antibodies used throughout the study

**Name**	**Source**	**Identifier**
Human reactive inflammasome antibody sampler kit II	Cell Signaling Technology	25620

Supplemental table 5: primer sequences used throughout the study

**Target gene**	**Forward (5'-3')**	**Reverse (5'-3')**
AIM2	CTGCTTAGACCAGTTGGCTTG	AGCTGACATCTGGAGTTCATAGC
ACTB	CACCATGTACCCTGGCATTG	AGTACTTGCGCTCAGGAGG
ASC	AAGCCAGGCCTGCACTTTAT	CTGGTACTGCTCATCCGTCA
CASP1	CCACAATGGGCTCTGTTTTT	CATCTGGCTGCTCAAATGAA
GAPDH	GGCTGCTTTTAACTCTGGTA	CTTGACGGTGCCATGGAATT
NLRC4	GAACCCTGTGACCTTGAAGA	TCAAGAATGCTCAGTTTGACCA
NLRP1	GATGAGACTCTGGTGTGGGA	GCACTAGTATCTCCTGGCGT
NLRP3	CCCAGGGATGAGAGTGTTGT	CAAGGAGATGTCGAAGCAGC
IFI16	TGACCACAATCAACTGTGAGG	CCTGGTCTTGATGACCTTGA
IL1B	CTGAAAGCTCTCCACCTCCA	CCAAGGCCACAGGTATTTTG
MTDN1	CAAAGGCCCCAACGTTGTAG	CGGGTTTTAGGGGCTCTTTG

Supplemental table 6: laboratory equipment used throughout the study

**Name**	**Source**	**Identifier**
Electrophoresis system	Bio-Rad, Deutschland	Mini-Protean Tetra System
EVOS microscope (brightfield)	ThermoFisher Scientific	XLCore
Gel Doc Imaging System	Vilber Lourmat, France	Fusion-SL 3500_WL
Keyence all-in-one microscope (fluorescence)	Keyence Deutschland GmbH	BZ-X810
TECAN Spark Multiplate Reader	TECAN Deutschland GmbH	N/A
TECAN infinite	TECAN Deutschland GmbH	F200PRO
PCR Thermocycler	Peqlab, Deutschland	Peqstar 2X
PCR Thermocycler	Applied Biosystems	StepOnePlus

Supplemental table 7: Patient and control characteristics (incl. healthy)

Characteristic	AAA<br>N = 93<br><br>*Median (Range)*	Non-AAA<br>N = 89<br><br>*Median (Range)*	Healthy<br>N = 10<br><br>*Median (Range)*	*P*-value AAA vs non-AAA	*P*-value AAA vs Healthy	*P*-value Healthy vs non-AAA
age (years)	70<br>(46-88)	71<br>(29-85)	56.5<br>(42-69)	0.8227	<0.0001	<0.0001
Sex						
female	18<br>(19.35%)	27<br>(30.34%)	5<br>(50%)	0.1214	0.0420	0.2851
male	75<br>(80.65%)	62<br>(69.66%)	5<br>(50%)			

AAA: abdominal aortic aneurysm; Statistics: Mann Whitney tests

Supplemental table 8: Spearman correlation analysis of cfDNA amounts and clinical parameters

**dsDNA in Plasma (ng/ml)**	**r**	**95% CI**	***P*-value**
Age (years)	-0.04831	-0.1966 to 0.1021	0.5172
BMI (kg/m^2)	0.1193	-0.03096 to 0.2643	0.1087
Max. AAA diameter	0.1962	-0.01397 to 0.3897	0.0595
WBC (cells/µl)	-0.1098	-0.2569 to 0.04230	0.1447
Cholesterol (mg/dl)	-0.2141	-0.3593 to -0.05889	**0.0057**
Creatinine (mg/dl)	-0.05355	-0.2033 to 0.09861	0.4777
Urea	-0.1166	-0.2633 to 0.03539	0.1212
CRP	-0.001621	-0.2134 to 0.2103	0.9878

**ssDNA in Plasma (ng/ml)**	**r**	**95% CI**	***P*-value**
Age (years)	0.1424	-0.008321 to 0.2868	0.0566
BMI (kg/m^2)	0.03722	-0.1139 to 0.1867	0.6198
Max. AAA diameter	0.2126	0.001946 to 0.4051	**0.0419**
WBC (cells/µl)	-0.09975	-0.2482 to 0.05329	0.1878
Cholesterol (mg/dl)	-0.1684	-0.3181 to -0.01048	**0.0317**
Creatinine (mg/dl)	0.08017	-0.07294 to 0.2296	0.2902
Urea	0.03738	-0.1155 to 0.1885	0.6224
CRP	0.09983	-0.1144 to 0.3052	0.3465

**mtDNA in Plasma (ng/ml)**	**r**	**95% CI**	***P*-value**
Age (years)	0.02626	-0.1199 to 0.1713	0.7177
BMI (kg/m^2)	-0.006282	-0.1558 to 0.1435	0.9329
Max. AAA diameter	-0.1569	-0.3548 to 0.05443	0.1331
WBC (cells/µl)	-0.02067	-0.1715 to 0.1311	0.7842
Cholesterol (mg/dl)	-0.01550	-0.1723 to 0.1421	0.8434
Creatinine (mg/dl)	0.08503	-0.06720 to 0.2334	0.2591
Urea	0.1687	0.01776 to 0.3121	**0.0244**
CRP	0.1342	-0.07996 to 0.3365	0.2048

r: spearman coefficient; BMI: body mass index; WBC: white blood cells; CRP: C-reactive protein; CI: confidence interval; Statistics: non-parametric spearman correlation

Supplemental table 9: Univariate analysis (binary logistic regression) of AAA presence.

Parameter	*P*-value	Odds Ratio	95% CI lower value	95% CI upper value
**ssDNA in plasma**	**0.0098**	**2.191**	**1.297**	**4.296**
**dsDNA in plasma**	**0.0348**	**30.09**	**2.013**	**1163**
**mtDNA copy number in plasma**	**0.0216**	**1.000**	**1.000**	**1.000**
**mtDNA copy number in PBMC**	**0.0016**	**1.010**	**1.004**	**1.017**
**Peripheral artery disease (PAD)**	**<0.0001**	**0.1046**	**0.047**	**0.216**
**COPD**	**0.0365**	**3.453**	**1.167**	**12.66**
**Diabetes mellitus**	**0.0210**	**0.4573**	**0.2322**	**0.8816**
Antihypertensive therapy	0.5771	1.316	0.5175	3.429
Hyperlipidaemia	0.5784	0.7790	0.3154	1.874
Ever smoked	0.1507	1.550	0.8675	2.883
age	0.7272	1.006	0.9734	1.004
sex	0.1394	1.691	0.8474	3.434

CI, confidence interval; COPD, chronic obstructive pulmonary disease; DNA, deoxyribonucleic acid; ss, single strand, ds, double strand; mt, mitochondrial

Supplemental table 10: Multivariate logistic regression of AAA presence including the mtDNA copy number in plasma and co-morbidities

Parameter	Sign. Different from zero *P*-value	Odds ratio	95% CI lower value	95% CI upper value
Amount of mtDNA in Plasma	0.0096	1.000	1.000	1.000
PAD	<0.0001	0.076	0.029	0.172
COPD	0.0135	5.912	1.578	27.43
Diabetes mellitus	0.1072	0.5317	0.244	1.143

CI, confidence interval; COPD, chronic obstructive pulmonary disease; DNA, deoxyribonucleic acid; mt, mitochondrial. P values for regression coefficients were calculated by Wald tests.

Supplemental table 11: Multivariate logistic regression of AAA presence including the mtDNA copy number/cell in PBMC and co-morbidities

Parameter	Sign. Different from zero *P*-value	Odds ratio	95% CI lower value	95% CI upper value
Amount of mtDNA in PBMC	0.0117	1.009	1.002	1.017
PAD	<0.0001	0.09107	0.037	0.202
COPD	0.0246	5.021	1.335	23.08
Diabetes mellitus	0.1578	0.5636	0.2521	1.248

CI, confidence interval; COPD, chronic obstructive pulmonary disease; DNA, deoxyribonucleic acid; mt, mitochondrial. P values for regression coefficients were calculated by Wald tests.

Supplemental Methods:

Isolation of chromatin from white blood cells

Chromatin, containing nucleosomes and DNA-binding proteins, was isolated from whole blood using ACK lysis buffer (Elabscience,# CK-A105) and a commercially available chromatin extraction kit (abcam, ab117152), by following the instructions of the manufacturer. Briefly, 2 ml of ACK lysis working buffer was added to 100 µl of fresh drawn blood and mixed thoroughly. After cooling to 4°C for 10 min., the mixture was centrifuged at 300 g for 5 min. and the supernatant was discarded. The pellet was resuspended in two ml PBS and centrifuged again at 300 g for 5 min. The resulting pellet, containing white blood cells, was resuspended in PBS and cells were counted. For chromatin extraction, 1 to 2 x 10^6 white blood cells were washed with 10 ml PBS twice by centrifugation for 5 min. and 100 µl of working lysis buffer (included in the abcam kit) per 10^6 cells was added to the final pellet. The resulting cell suspension was transferred to a new vial, incubated on ice for 10 min., vortexed vigorously for 10 sec. and centrifuged at 5000 rpm for 5 min in a microliter centrifuge. After removing the supernatant, the Working extraction buffer (included in the abcam kit) was added to the chromatin pellet and resuspended by pipetting up and down. Samples were incubated on ice for 10 min. and vortexed occasionally. To increase chromatin extraction, samples were sonicated 2 x 20 sec. and allowed to cool on ice in between. Extracts were centrifuged at 12000 rpm at 4°C for 10 min. and the supernatant was transferred to a new vial before Chromatin Buffer (included in the abcam kit) was added at a 1:1 ratio. Chromatin solutions were snap frozen in liquid nitrogen and stored at -80°C until further use.

For stimulation experiments, 2 µl of the chromatin extract, corresponding to ~40 µg, was added to 2 ml of cell culture medium in 6-well plates.

Supplemental Figure S1

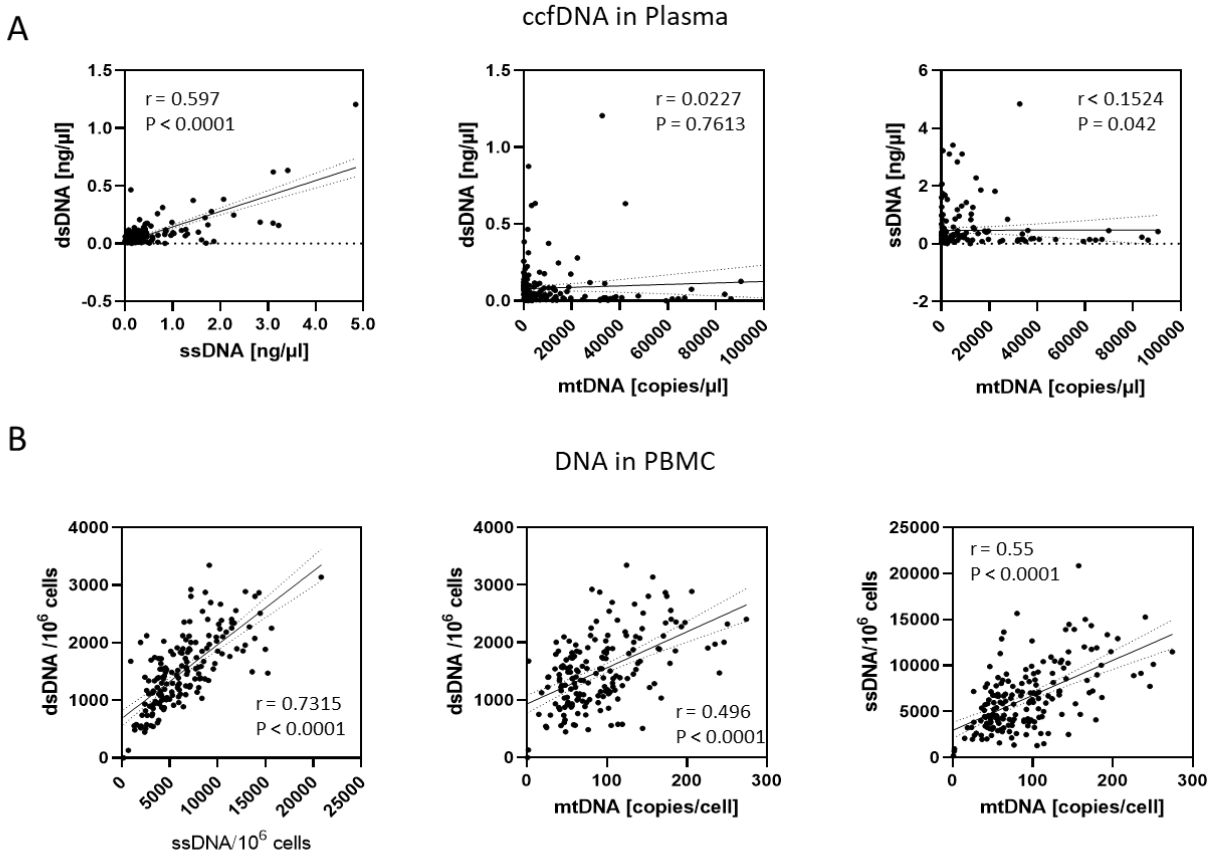

Spearman correlation analysis to determine the relationship between the DNA amounts in plasma or PBMC and clinical parameters. A. Amount of dsDNA versus ssDNA, dsDNA versus mtDNA copy number, and ssDNA versus mtDNA copy number in the plasma of study participants. **B**. Amount of dsDNA versus ssDNA, dsDNA versus mtDNA copy number/cell and ssDNA versus mtDNA copy number/cell in PBMC of study participants. r: Spearman correlation coefficient.

Supplemental Figure S2

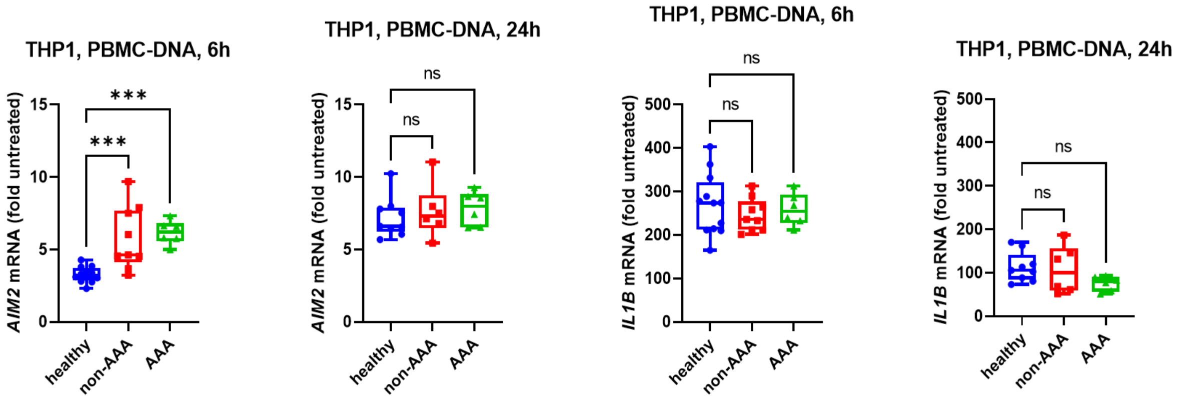

Relative gene expression of *AIM2* and *IL1B* in THP-1 cells. THP-1 cells were grown in RPMI-1640 (ThermoFisher Scientific, Gibco), supplemented with 10% FBS (ThermoFisher Scientific, Gibco) and 1% Penicillin/Streptomycin at 37°C, 5% CO2 in a humidified atmosphere. For differentiation, cells were treated with 20 nM phorbol12-myristate-13-acetate (PMA) for 48 hours before the experiments. On the day of the experiment, cells were shifted to RPMI-1640 medium without phenol red and primed with LPS (100 ng/ml) or left unprimed. For stimulation, 50 ng/ml PBMC-DNA from different donors (n = 4 healthy, n = 3 non-AAA, n = 3 AAA) was added to 1 x 10^6 cells. Cells were harvested and lysed in RLT buffer 6h and 24h after stimulation and mRNA was isolated and analysed by real-time PCR as described in the main text. Data were statistically analyzed by Oridnary on-way ANOVA and Sidak‘s or Dunnett‘s multiple comparison tests.

Supplemental Figure S3

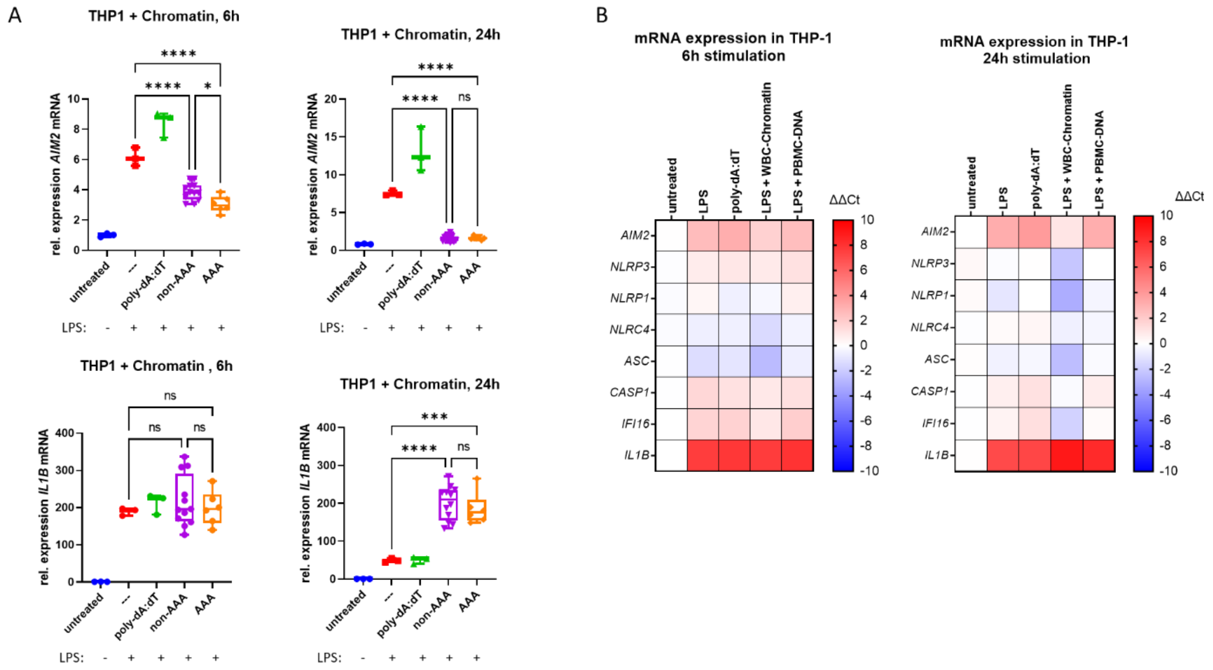

Relative gene expression of inflammasome genes in THP-1 cells after stimulation with different cfDNA types. A. *AIM2* and *IL1B* expression in untreated THP-1 cells (n = 3), in response to stimulation with LPS (n = 3), or in response to LPS + poly dAdT (n = 3), LPS + chromatin from non-AAA patients (n = 9) or LPS + Chromatin from AAA patients (n = 6). B. Heatmap of the median expression levels of eight inflammasome genes in THP-1 cells after stimulation. Data were statistically analyzed by Oridnary on-way ANOVA and Sidak‘s multiple comparison test.

Supplemental Figure S4

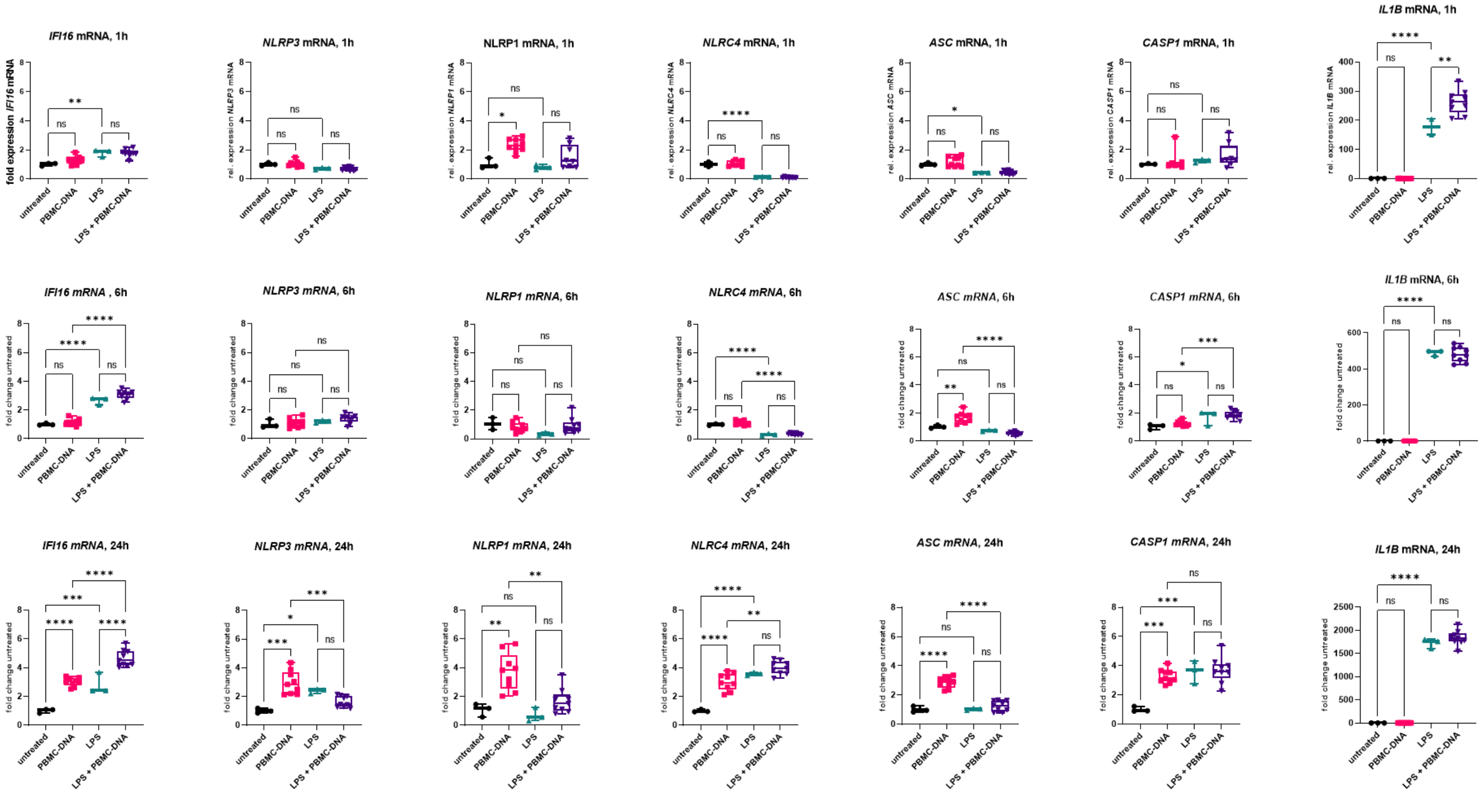

Inflammasome activation by PBMC-DNA in THP-1. THP-1 cells were differentiated with 20 nM phorbol-12-myristate-13-acetate (PMA) for two days and subsequently stimulated with vehicle, or 50 ng/ml PBMC-DNA (n = 3) AAA-patients, in the presence or absence of 100 ng/ml LPS. Cells were incubated for 1h, 6h or 24 hours as indicated. Relative expression of mRNAs was determined by RT-qPCR. Data were derived from three biological replicates, analysed in triplicate. Statistical analysis was performed by ordinary one-way ANOVA with subsequent Šídák’s multiple comparison test.

Supplemental Figure S5

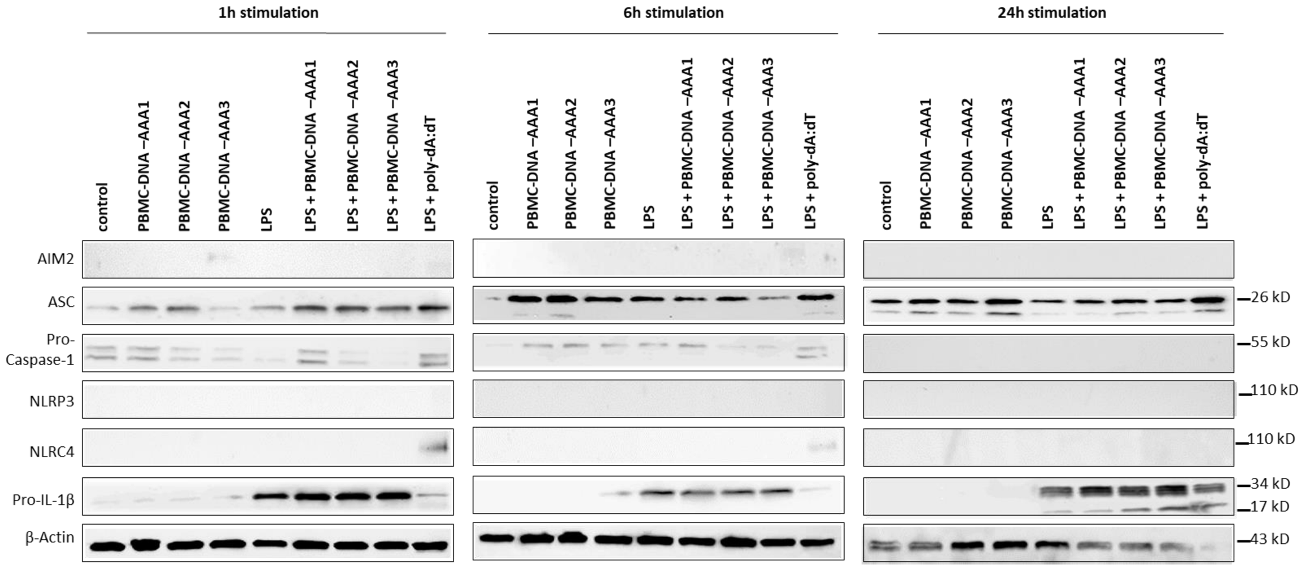

Inflammasome protein expression induced by PBMC-DNA in THP-1. Representative immunoblot analysis of inflammasome protein expression in cell lysates after treatment are shown. THP-1 cells were differentiated with 20 nM phorbol-12-myristate-13-acetate (PMA) for two days and subsequently stimulated with vehicle, or 50 ng/ml PBMC-DNA (n = 3) AAA-patients or 1 µg/ml poly(dA:dT)/LyoVec, in the presence or absence of 100 ng/ml LPS (6h or 24h as indicated). For shorter stimulation, PMA-differentiated cells were left untreated or primed for 2h with 200 ng/ml LPS, followed by 1h stimulation with vehicle, or 50 ng/ml PBMC-DNA (n = 3) AAA-patients.

Supplemental Figure S6

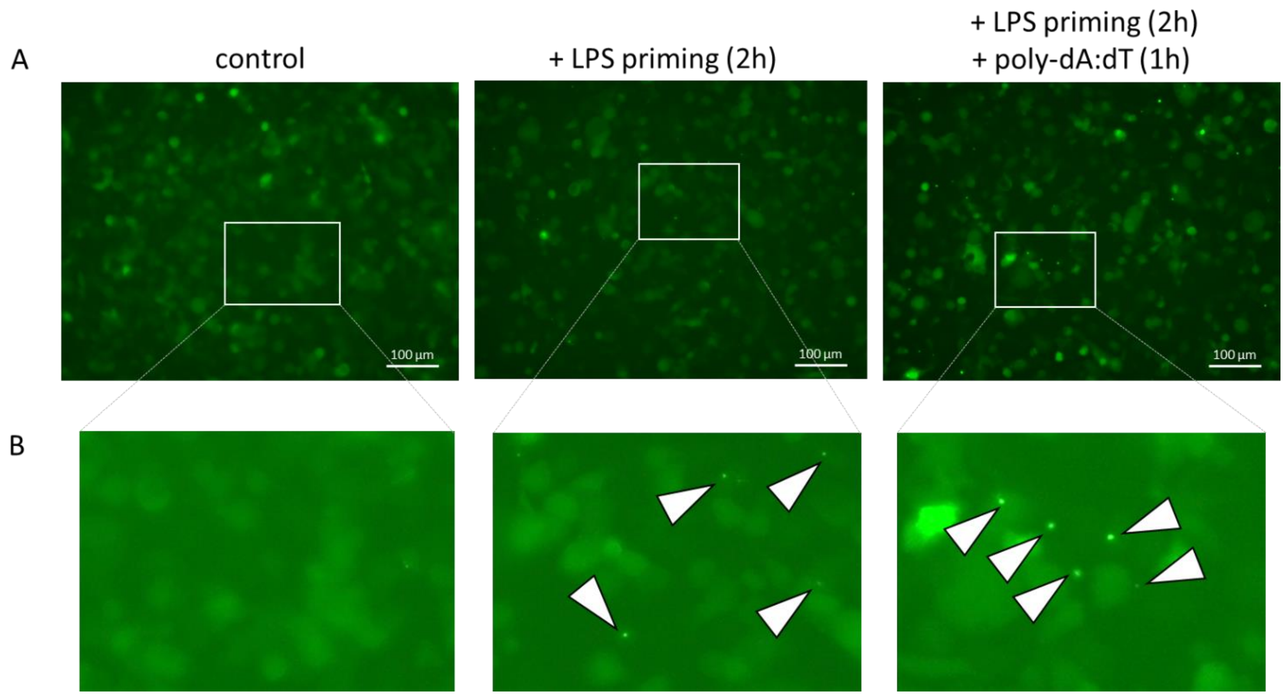

Inflammasome activation by PBMC-DNA in THP-1-ASC-GFP cells. A) Representative fluorescence micrographs of ASC Specks in THP-1-ASC-GFP. THP-1-ASC-GFP cells were differentiated with 20 nM phorbol-12-myristate-13-acetate (PMA) for two days, primed for 2h with 200 ng/ml LPS or vehicle, and stimulated for 1h with vehicle or 100 ng/ml poly-dA:dT. Images were taken with an original magnification of 200x. B). Enlarged section of the images shown in A. The images are shown at maximum brightness to make all cells visible. White arrows point to ASC specks, representing inflammasomes.

Supplemental Figure S7

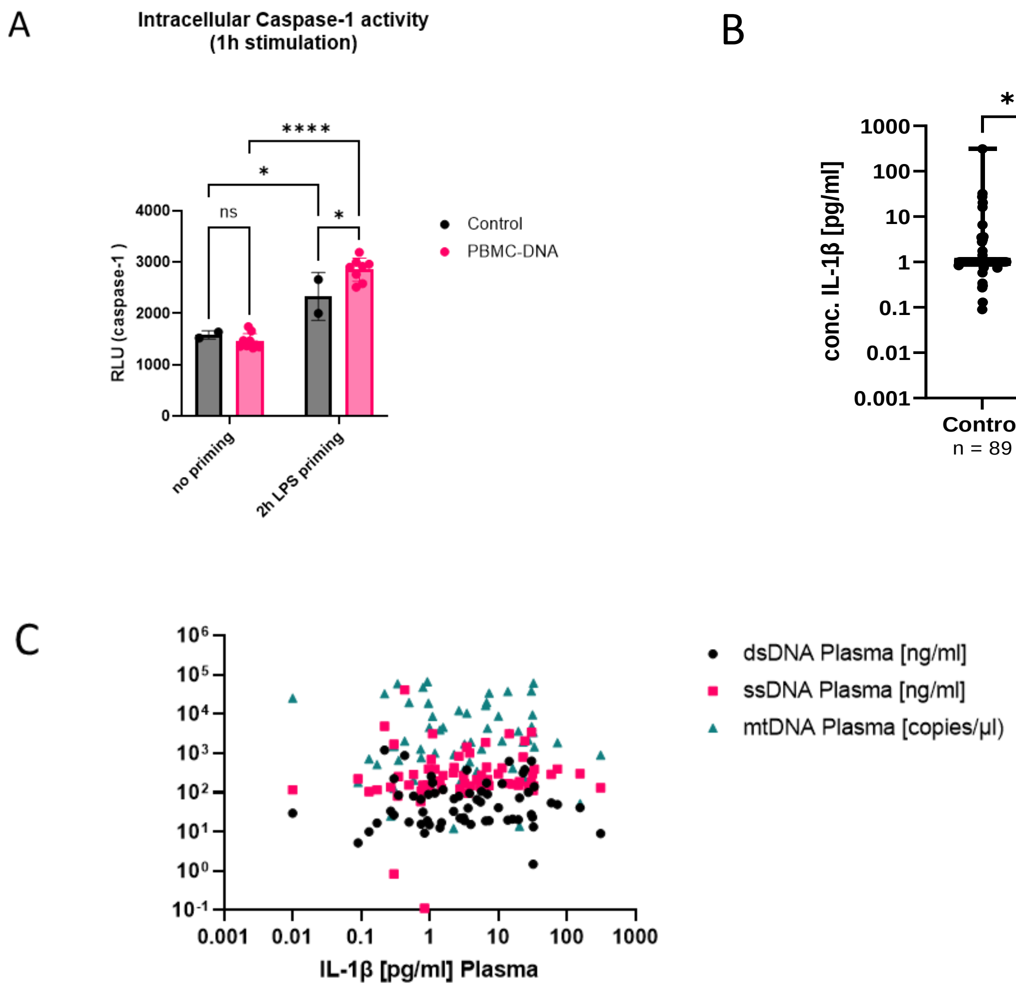

	IL-1b [pg/ml] Plasma vs. dsDNA Plasma [ng/ml]	IL-1b [pg/ml] Plasma vs. ssDNA Plasma [ng/ml]	IL-1b [pg/ml] Plasma vs. mtDNA Plasma [copies/µl]
Spearman r			
r	0.1429	0.07229	0.04015
95% confidence interval	-0.007373 to 0.2868	-0.07867 to 0.2200	-0.1106 to 0.1891
P value			
P (two-tailed)	0.0550	0.3335	0.5915
P value summary	ns	ns	ns
Exact or approximate P value?	Approximate	Approximate	Approximate
Significant? (alpha = 0.05)	No	No	No
Number of XY Pairs	181	181	181

A. Intracellular caspase-1 activity in stimulated THP-1 cells. THP-1 cells were differentiated with 20 nM phorbol-12-myristate-13-acetate (PMA) for two days and subsequently primed for 2 h with 200 ng/ml LPS. Next, cells were stimulated with vehicle, or 50 ng/ml PBMC-DNA (n = 3) from AAA-patients for 1 hour. Intracellular caspase-1 activity was determined by luminescence-based Caspase-Glo 1 Inflammasome assay, following the instructions of the manufacturer (Promega, Walldorf, Germany, # G9951).
B. Comparison of Il-1β levels in plasma of n = 89 control and n = 93 AAA patients. Il-1β was quantified by ELISA using Human IL-1β Mini TMB ELISA Development Kit and TMB ELISA Buffer Kit (PeproTech) following the instructions of the manufacturer. Statistics: Mann-Whitney test with a two-tailed P-value. Note that only values above 0.0 are shown as dots.
C. Spearman correlation analysis to determine the relationship between the IL- 1β levels and cfDNA levels in the plasma of n = 181 patients. r = Spearman correlation coefficient
