# Supplementary material for: Exploring circulating cell-free DNA as a biomarker and as an inducer of AIM2-inflammasome-mediated inflammation in patients with abdominal aortic aneurysm
Source: Sci Rep. 2025 Jun 20;15:20196. doi: 10.1038/s41598-025-06220-5 (PMC12181305; doi:10.1038/s41598-025-06220-5)

Uncrapped Western Blots,

1 h stimulations, corresponding to supplemental figure S5 and Figure 4c

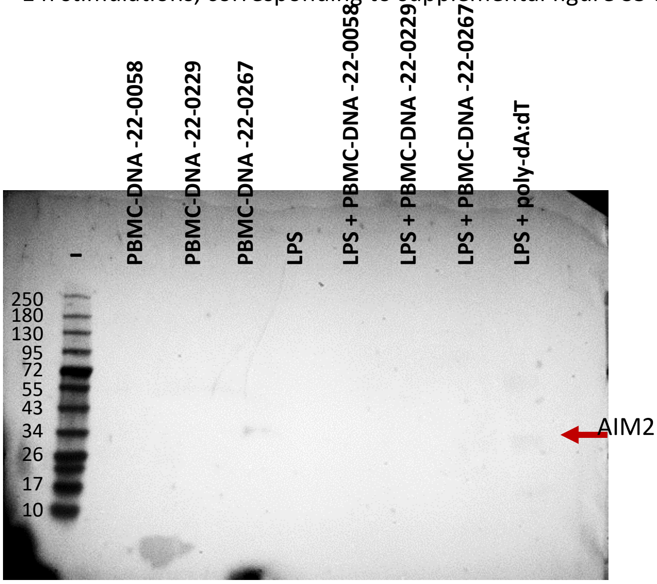

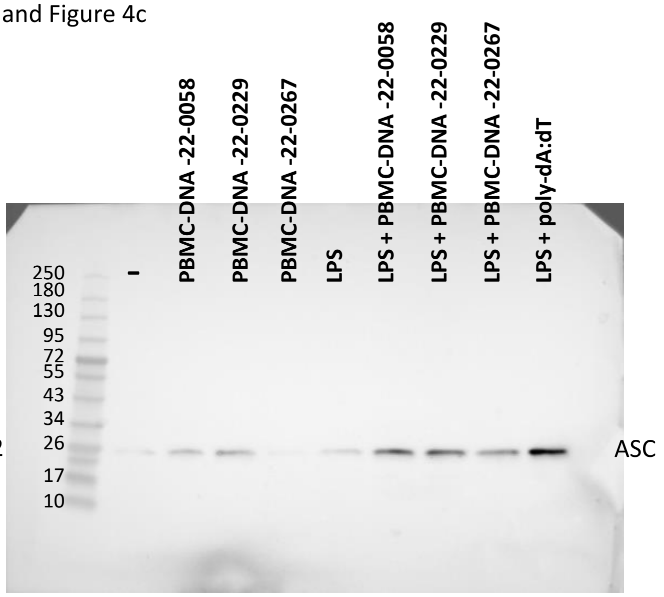

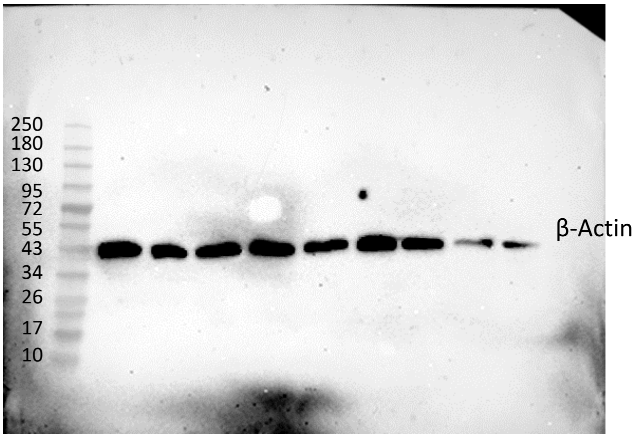

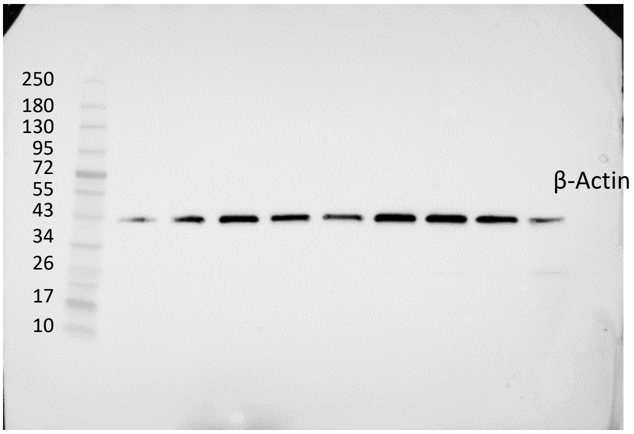

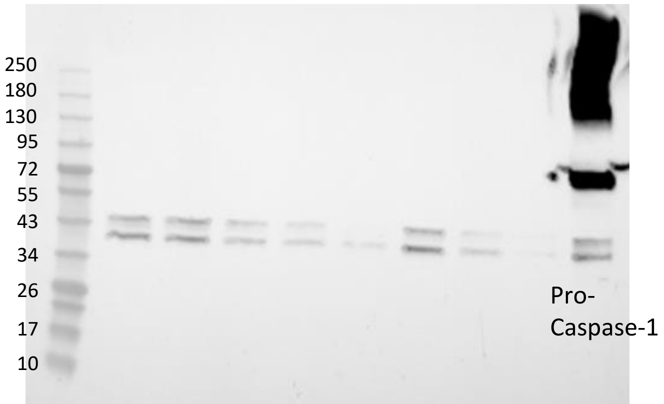

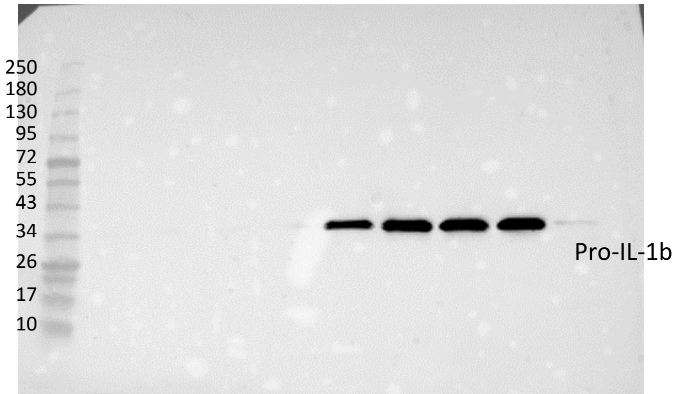

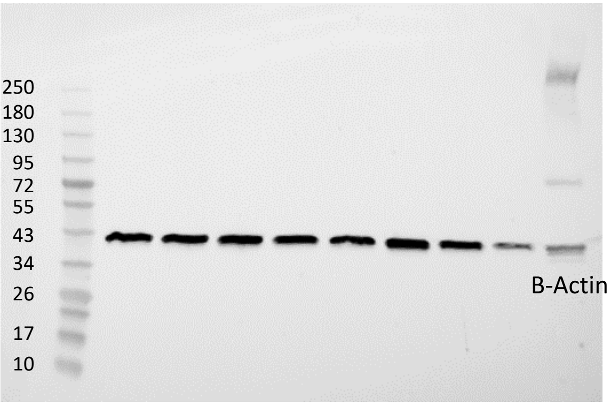

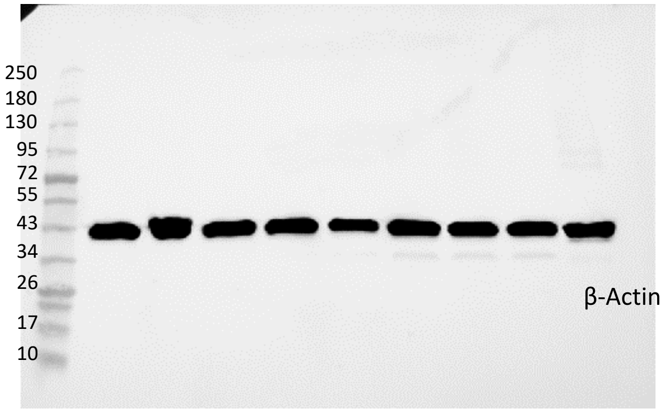

Uncrapped Western Blots

1 h stimulations, corresponding to supplemental figure S3

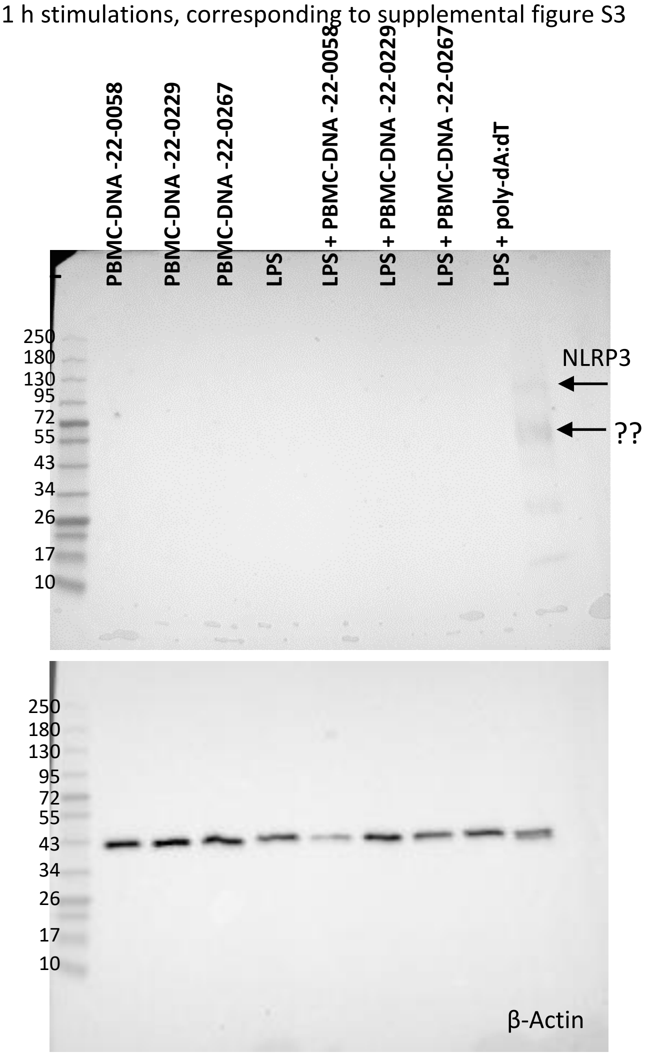

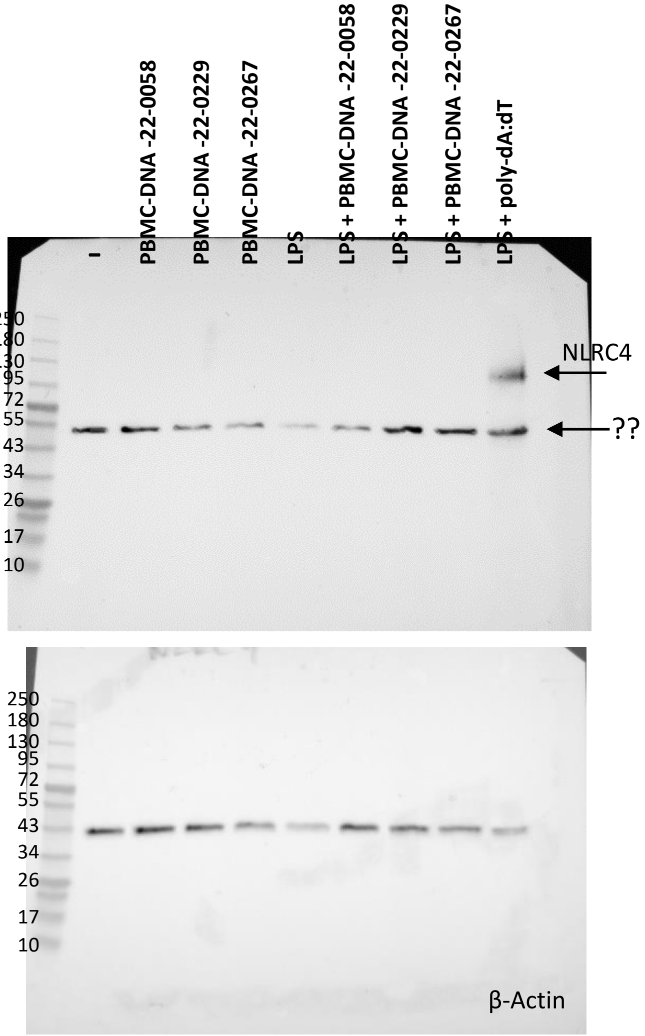

Uncrapped Western Blots

6 h stimulations, corresponding to supplemental figure S5 and Figure 4c

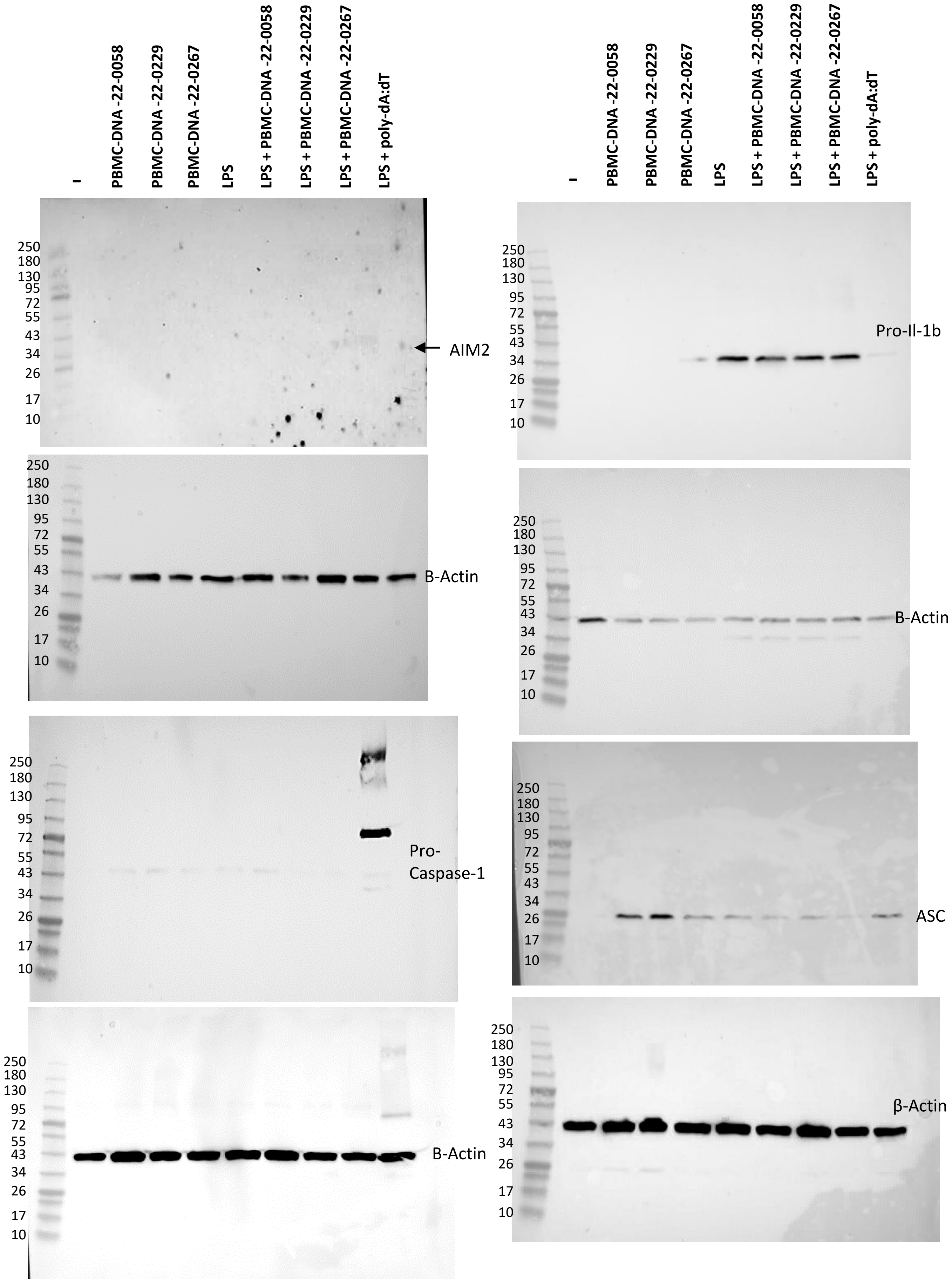

Uncrapped Western Blots

6 h stimulations, corresponding to supplemental figure S5

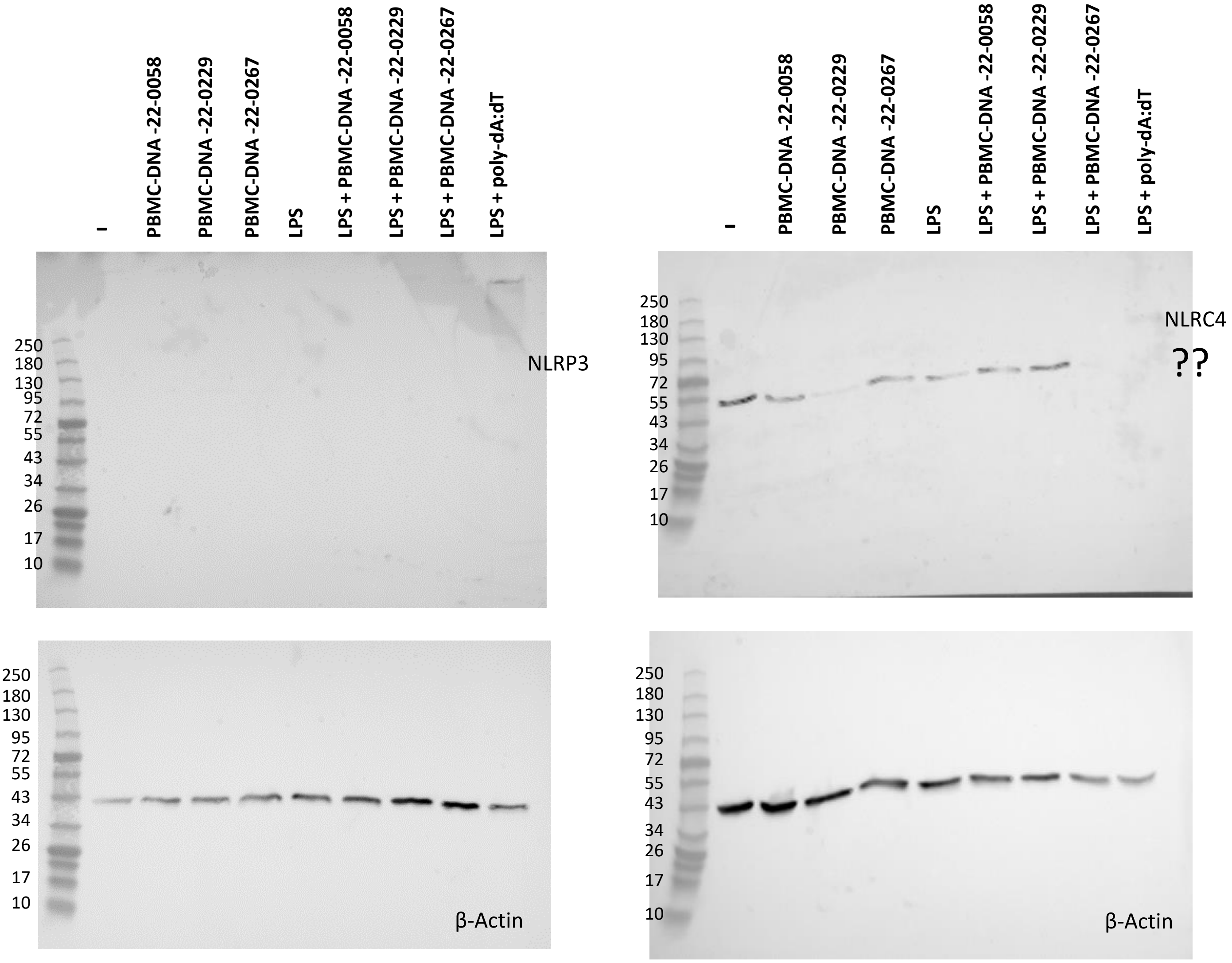

Uncrapped Western Blots

24 h stimulations, corresponding to supplemental figure S5 and Figure 4c

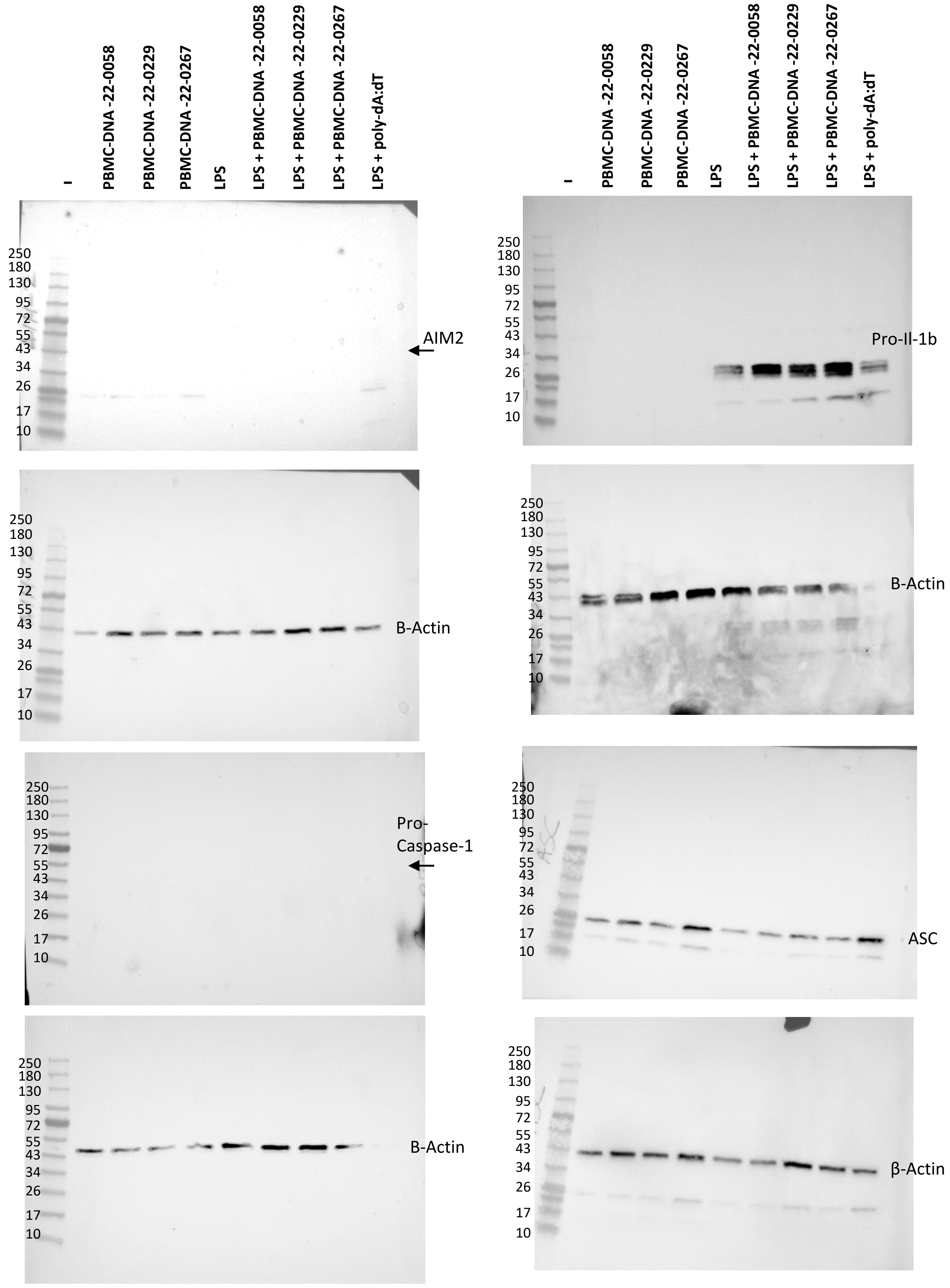

Uncrapped Western Blots

6 h stimulations, corresponding to supplemental figure S5

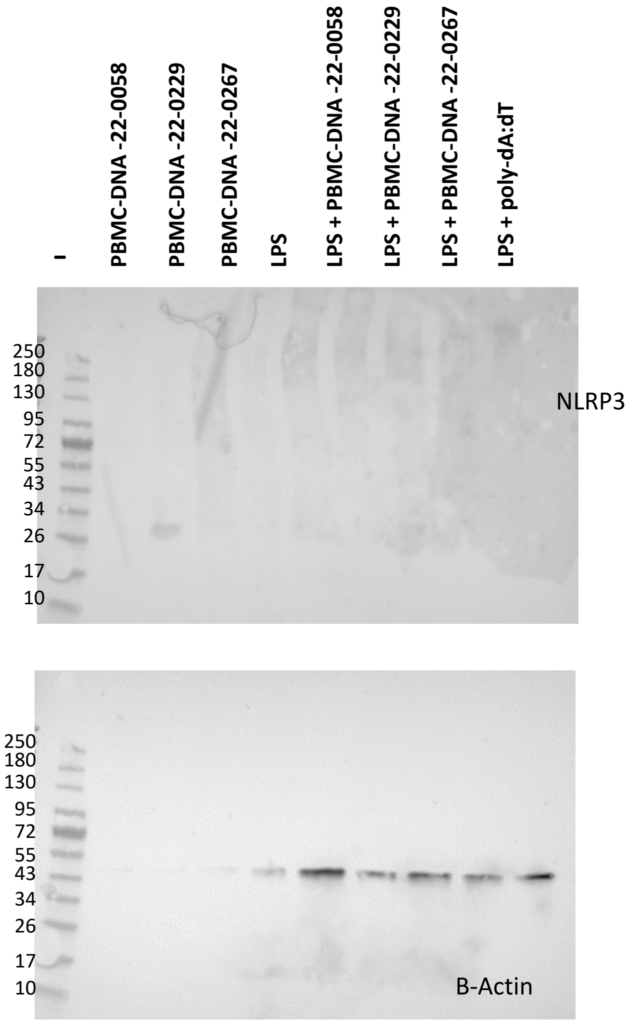

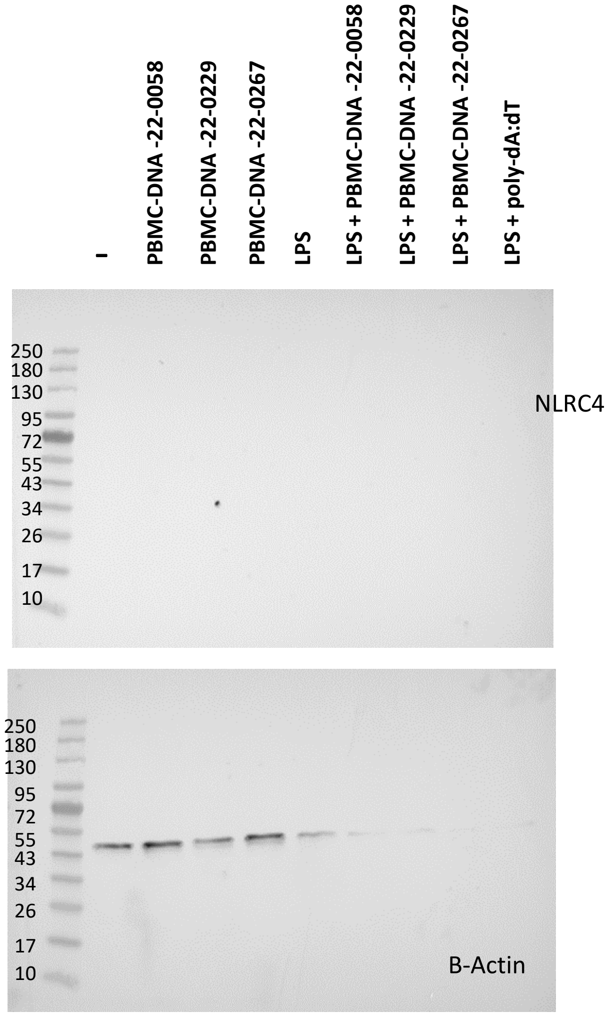

Supplement: Supplementary file 2 — Supplementary Information 2. [file 41598_2025_6220_MOESM2_ESM.pdf]
